# Integration of spatial and single-cell data across modalities with weakly linked features

**DOI:** 10.1038/s41587-023-01935-0

**Published:** 2023-09-07

**Authors:** Shuxiao Chen, Bokai Zhu, Sijia Huang, John W. Hickey, Kevin Z. Lin, Michael Snyder, William J. Greenleaf, Garry P. Nolan, Nancy R. Zhang, Zongming Ma

**Affiliations:** 1 Department of Statistics and Data Science, The Wharton School, University of Pennsylvania, Philadelphia, PA USA; 2 Department of Microbiology and Immunology, Stanford University, Stanford, CA USA; 3 Department of Pathology, Stanford University, Stanford, CA USA; 4 Department of Biostatistics, University of Washington, Seattle, WA USA; 5 Department of Genetics, Stanford University, Stanford, CA USA; 6 Department of Statistics and Data Science, Yale University, New Haven, CT USA

**Keywords:** Data integration, Statistical methods

## Abstract

Although single-cell and spatial sequencing methods enable simultaneous measurement of more than one biological modality, no technology can capture all modalities within the same cell. For current data integration methods, the feasibility of cross-modal integration relies on the existence of highly correlated, a priori ‘linked’ features. We describe matching X-modality via fuzzy smoothed embedding (MaxFuse), a cross-modal data integration method that, through iterative coembedding, data smoothing and cell matching, uses all information in each modality to obtain high-quality integration even when features are weakly linked. MaxFuse is modality-agnostic and demonstrates high robustness and accuracy in the weak linkage scenario, achieving 20~70% relative improvement over existing methods under key evaluation metrics on benchmarking datasets. A prototypical example of weak linkage is the integration of spatial proteomic data with single-cell sequencing data. On two example analyses of this type, MaxFuse enabled the spatial consolidation of proteomic, transcriptomic and epigenomic information at single-cell resolution on the same tissue section.

## Abstract (web summary)

MaxFuse enables data integration between modalities even when features are weakly correlated.

## Main

Recent technological advances have enabled analyses of the proteome and metabolome^1,2^, transcriptome^3^ and various aspects of the epigenome such as methylation^4^, histone modification^5–7^ and chromatin accessibility^5,8^ within individual cells. In addition to technologies operating on dissociated single cells, rapid progress has been made on the in situ measurement of transcriptome^9^, proteome^10–14^, epigenome^15^ and other modalities on tissue sections at single-cell or close to single-cell resolution, retaining the spatial context. To harness the new technologies and growing data resources for biological discovery, a primary challenge is the reliable integration of data across modalities. Cross-modal integration, also referred to as ‘diagonal integration’^16,17^, is the alignment of single cells or spatial spots across datasets where different features (or modalities) are profiled in each dataset. This cross-modal integration step underpins many types of downstream analyses, and its importance is evident in the myriad methods that have already been developed to tackle such tasks^18–24^.

Despite the progress, key limitations still hinder reliable cross-modal integration, as highlighted by recent surveys^16,17,25^. A key factor limiting the accuracy of existing methods is the strength of linkage between modalities, as we define below. A feature is ‘linked’ between two modalities if it was measured in, or can be predicted by, both modalities. In the terminology of refs. ^16,17^, these linked features can serve as ‘anchors’ for integration. For example, to integrate single-cell assay for transposase-accessible chromatin sequencing (scATAC-seq) and single-cell RNA sequencing (scRNA-seq) data, most existing methods predict the ‘activity’ for each gene in each cell of the scATAC-seq data based on the accessibility of the gene’s surrounding chromatin; then, each gene’s ATAC activity can be ‘linked’ to its RNA expression, thus mapping cells from the two datasets into the same feature space. Similarly, between RNA and protein assays, the abundance of each protein can be ‘linked’ to the expression of its coding gene in the RNA assay.

Most existing methods are designed for scenarios where there is a large number of linked features that also exhibit strong cross-modality correlations, a situation that we refer to as ‘strong linkage’. For example, between scRNA-seq and scATAC-seq, every gene in the genome can be linked, and the correlation between gene activity and RNA expression is often high enough for enough genes to allow for precise integration^18,19,22^. To achieve strong linkage, some methods attempt to learn a mapping from the features of one modality to the features of the other modality through a ‘training set’ consisting of data obtained when both modalities are simultaneously observed in each cell/spot^23,26^. While this strategy may be applicable towards the integration of data from biological systems that are similar to the training set, it is questionable how well it can generalize to unseen systems.

Cross-modality integration in scenarios of weak linkage, where the number of linked features is small and/or the between-modality correlation for the linked features is weak, is especially challenging. A prototypical example of weak linkage is between targeted protein assays^14,27^ and transcriptome or epigenome assays such as scRNA-seq or scATAC-seq. Such scenarios are becoming extremely common as spatial proteomic technologies have been widely adopted^10–14^, and complementing RNA and ATAC sequencing to achieve more complete tissue characterization^28–31^. We will reveal, through comprehensive benchmarks, the limitations of existing state-of-the-art methods in such difficult cases.

To address these limitations, we developed a method that we call MaxFuse, a model-free, adaptable method that can accurately integrate data across weakly linked modalities. We systematically benchmarked the performance of MaxFuse across single-cell protein, RNA and chromatin accessibility multiome ground-truth datasets. Across a wide variety of datasets, MaxFuse has superior performance compared with other state-of-the-art integration methods. Although the largest improvements in accuracy were observed under weak linkage, under strong linkage MaxFuse was comparable to the current best method in integration performance with substantial improvement in speed.

We further demonstrate the analyses enabled by MaxFuse with two examples. First, in the integration of scRNA-seq and CODEX multiplexed in situ protein profiling data from the human tonsil, we showed that MaxFuse identified correct spatial gradients in the RNA expression of genes not included in the 46-marker protein panel. Second, MaxFuse was applied to an atlas-level integration of spatial proteomic and single-cell sequencing datasets^31^. We demonstrate how to perform tri-modal integration of CODEX, single-nucleus RNA sequencing (snRNA-seq) and single-nucleus ATAC sequencing (snATAC-seq) data that revealed spatial patterns of RNA expression and transcription factor binding site accessibility at single-cell resolution. We have implemented MaxFuse as a Python package which is freely available to the public at https://github.com/shuxiaoc/maxfuse.

## Results

### Cross-modality matching via iterative smoothed embedding

**Fig. 1:**
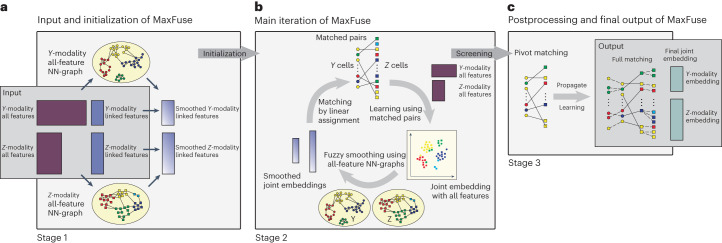
Overview of MaxFuse pipeline. **a**, The input consists of two pairs of matrices. The first pair consists of all features from each modality, and the second pair consists of only the linked features. MaxFuse uses all features within each modality to create a nearest-neighbor graph (that is, all-feature NN-graph) for cells in that modality. Fuzzy smoothing induced by the all-feature NN-graph is applied to the linked features in each modality. Cross-modal cell matching based on the smoothed linked features initializes the iterations in **b**. **b**, In each iteration, MaxFuse starts with a list of matched cell pairs. A cross-modal cell pair is called a pivot. MaxFuse learns canonical correlation analysis (CCA) loadings over all features from both modalities based on these pivots. These CCA loadings allow the computation of CCA scores for each cell (including cells not in any pivot), which are used to obtain a joint embedding of all cells across both modalities. For each modality, the embedding coordinates then undergo fuzzy smoothing based on the modality-specific all-feature NN-graphs (obtained in **a**). Next, the smoothed embedding coordinates are supplied to a linear assignment algorithm that produces an updated list of matched pairs to start the next iteration. **c**, After iterations end, MaxFuse screens the final list of pivots to remove low-quality matches. The retained pairs are called refined pivots. Within each modality, any cell that is not part of a refined pivot is connected to its nearest neighbor that belongs to a refined pivot and is matched to the cell from the other modality in this pivot. This propagation step results in a full matching. MaxFuse further learns the final CCA loadings over all features from both modalities based on the refined pivots. The resulting CCA scores give the final joint embedding coordinates.

The input to MaxFuse are data from two modalities in the form of two pairs of matrices (Fig. 1a). For convenience, we can call the two modalities *Y* and *Z*. First, we have a pair of cell-by-feature matrices that contain all measured features in each modality. In addition, we represent the initial knowledge about the linkage between the two modalities as another pair of cell-by-feature matrices whose columns have one-to-one correspondences. To distinguish between these two pairs of matrices, we call the former all-feature matrices and the latter linked-feature matrices. For example, when one modality is protein abundance over a small antibody panel and the other is RNA expression over the whole transcriptome, the two all-feature matrices have drastically different numbers of columns, one being the number of proteins in the panel and the other being the number of genes in the transcriptome; the linked-feature matrices, on the other hand, have an equal number of columns, where each column in the protein matrix is one protein and its corresponding column in the RNA linked-feature matrix is its coding gene. When the number of cells is large, we recommend aggregating cells with similar features into meta-cells, as described in the Methods, before applying MaxFuse. In that case, each row in the above matrices would represent a meta-cell. The procedure below does not depend on whether single cells or meta-cells are used, and thus we will refer to each row as a ‘cell’.

During stage 1 of the MaxFuse pipeline, cell–cell similarities are identified within each modality and initial cross-modal matching of cells is performed. This stage consists of three major steps (Fig. 1a). In step 1, for each modality, we use all features to compute a fuzzy nearest-neighbor graph connecting all cells measured in that modality. This graph, by utilizing the information in all features, provides the best possible summary of the cell–cell similarity for the given modality. In particular, cells that are close in this graph should have comparable values for their linked features. Thus, in step 2, MaxFuse boosts the signal-to-noise ratio in the linked features within each modality by shrinking their values, for each cell, towards the cell’s graph-neighborhood average. We call this step ‘fuzzy smoothing’. In step 3, MaxFuse computes distances between all cross-modal cell pairs based on the smoothed, linked features and applies linear assignment^32^ on the cross-modal pairwise distances to obtain an initial matching of cells. The initial matching serves as the starting point for stage 2.

Stage 2 of MaxFuse improves cross-modal cell matching quality by iterating the sequence of joint embedding, fuzzy smoothing and linear assignment steps (Fig. 1b). Starting with the initial matches obtained in stage 1, in each iteration, MaxFuse first learns a linear joint embedding of cells across modalities by computing a canonical correlation based on all features of the cross-modal matched cell pairs. Then, coordinates of this joint embedding are treated as new linked features of each modality and fuzzy smoothing is applied on them based on the all-feature nearest-neighbor graphs computed in stage 1. Finally, MaxFuse updates the cell-matching across modalities by applying linear assignment on the pairwise distances of these fuzzy-smoothed joint embedding coordinates. The resulting matching is used to start the next iteration. Matching quality improves with each iteration until available information in all features, and not just the linked features, has been used.

In stage 3, MaxFuse processes the last cross-modal cell matching from stage 2 and produces final outputs. First, MaxFuse screens the matched pairs from the last iteration, retaining high-quality matches as pivots. The pivots are used in two complementary ways: (1) they are used one last time to compute a final joint embedding of all cells in both modalities; (2) for any unmatched cell in either modality, its closest neighbor within the same modality that belongs to a pivot is identified and, as long as its distance to this neighbor is below a threshold, the match in the pivot is propagated to the cell. Thus, the final output of MaxFuse has two components: (1) a list of matched pairs across modalities, and (2) a joint embedding of all cells in both modalities. See the Methods for more MaxFuse algorithm details.

### Integration of transcriptome and targeted protein data

We benchmarked MaxFuse on a cellular indexing of transcriptomes and epitopes sequencing (CITE-seq) dataset^33^ that included measurements of 228 protein markers and whole transcriptome on peripheral blood mononuclear cells (PBMCs). For comparison, we also applied four state-of-the-art integration methods, Seurat (V3) (ref. ^24^), Liger^22^, Harmony^20^ and BindSC^34^, to this same dataset. Protein names were converted to RNA names manually to link the features between datasets. In each repetition of our experiment, we randomly subsampled 10,000 cells and applied all methods, and assessed using the benchmarking criteria to be described below. We performed five such repetitions and averaged the criteria across repetitions. For all integration methods, we masked the known cell–cell matching between the protein and RNA modalities, and then used the known matching for assessment.

Methods were assessed using six different criteria that measure both cell-type-level label transfer accuracy as well as cell-level matching accuracy. Two criteria were used to judge cell-type-level label transfer accuracy. Cells were annotated at two levels of granularity (from ref. ^33^): level 1, which differentiates between eight major cell types; and level 2, a finer classification which differentiates between 31 cell types. The proportions of matched pairs that shared the same label at both annotation levels were reported, with higher proportions indicating higher matching quality. Two criteria assessed the quality of cross-modal joint embedding of cells. A high-quality joint embedding should preserve biological signal, as reflected by the separation of known cell types, while mixing the two modalities as uniformly as possible. Usually, there is a trade-off between these two goals. To aggregate quality assessments of biological signal preservation and modality mixing, we calculated *F*_1_ scores based on average silhouette width (slt_f1) and on adjusted Rand index (ari_f1), as proposed in ref. ^35^. For both criteria, higher *F*_1_ indicates a better embedding. The fifth criterion, Fraction Of Samples Closer Than True Match (FOSCTTM)^19,36,37^, was used to quantify the quality of joint embedding at single-cell resolution. For each cell, we computed the fraction of cells in the other modality that is closer than its true match in the joint embedding space. FOSCTTM is the average of this fraction over all cells in both modalities. The lower the value of this score, the closer the true matches are in the joint embedding, and, hence, the better the joint embedding. The last criterion is Fraction Of Samples whose true matches are among their *K*-Nearest Neighbors (FOSKNN) in the joint embedding space. For any given *k* ≥ 1, the higher this proportion, the better the joint embedding. For precise definitions of these criteria, see the Methods.

**Fig. 2:**
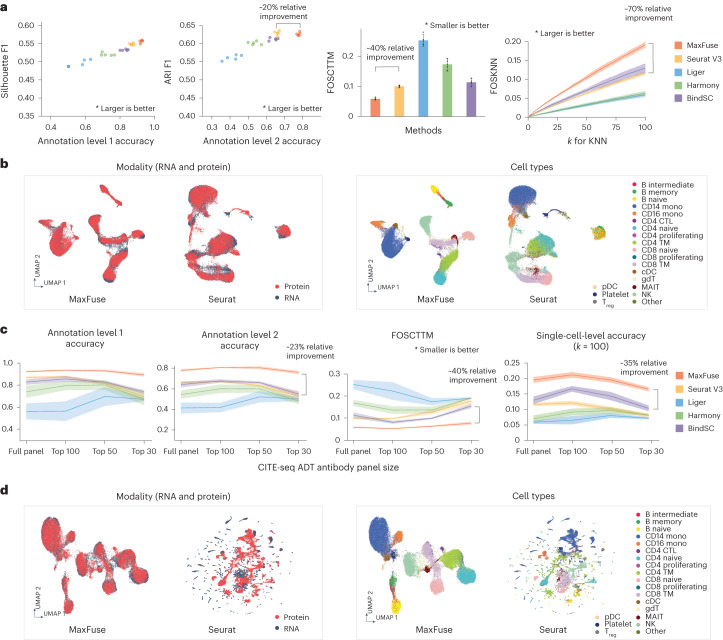
Benchmarking of MaxFuse and other integration methods on ground-truth CITE-seq PBMC data. **a**, Matching and integration performance of MaxFuse and other methods on CITE-seq PBMC dataset with the full antibody panel (228 antibodies). The barplot and the line plot show mean value with the error bar or shadow area covering 95% CI on both sides, from *n* = 5 randomly subsampled cell batches. **b**, UMAP visualization of MaxFuse and Seurat (V3) integration results of CITE-seq PBMC dataset with the full panel, colored by modality (left) or cell type (right). **c**, Matching and integration performance of MaxFuse and other methods on CITE-seq PBMC dataset with reduced antibody panels (full 228 antibodies or the most informative 100, 50 or 30 antibodies.) For each method, the line indicates mean value with the shadow area covering 95% CI on both sides, from *n* = 5 randomly subsampled cell batches. **d**, UMAP visualization of MaxFuse and Seurat (V3) integration results of CITE-seq PBMC dataset with the 30 most informative of the original 228 antibodies, colored by modality (left) or cell type (right). 95% CI, 95% confidence interval; cDC, classical dendritic cells; CTL, cytotoxic T lymphocytes; gDT, gamma delta T cells; KNN, *k*-nearest neighbors; MAIT, mucosal-associated invariant T cells; NK, natural killer cells; pDC, plasmacytoid dendritic cells; TM, T memory cells; T_reg_, T regulatory cells.

**Extended Data Fig. 1:**
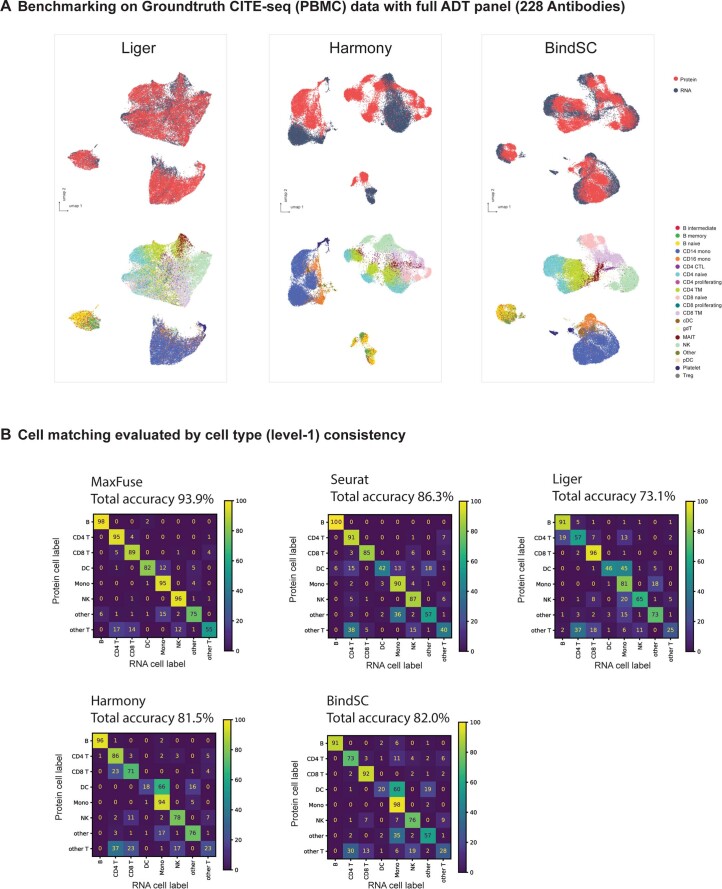
Benchmarking on ground-truth CITE-seq PBMC data with all 228 antibodies from Hao et al.^33^. **(A)** UMAP visualization of Liger, Harmony, and BindSC integration results, colored by modality (upper panel) or level 2 cell types (lower panel). **(B)** Cell matching accuracy results (cell type level 1) of different methods.

Based on all these criteria, MaxFuse was superior by a sizable margin (Fig. 2a). Importantly, MaxFuse resulted in accurate cell matching across weakly linked modalities (for example, level 1 accuracy 93.9%, better by over 7% in absolute scale than the second best method (Extended Data Fig. 1)). The Uniform Manifold Approximation and Projection (UMAP) plots calculated based on the postintegration embedding from respective methods (Fig. 2b and Extended Data Fig. 1), colored by modality and by level 2 cell-type annotation, showed that MaxFuse achieved both better mixing of the two modalities (left panel) and better preservation of biological signals (right panel). For example, a clearly resolved trajectory of B cell subtypes (B naive, intermediate and memory cells) was apparent after MaxFuse integration but not after integration by other methods.

**Extended Data Fig. 2:**
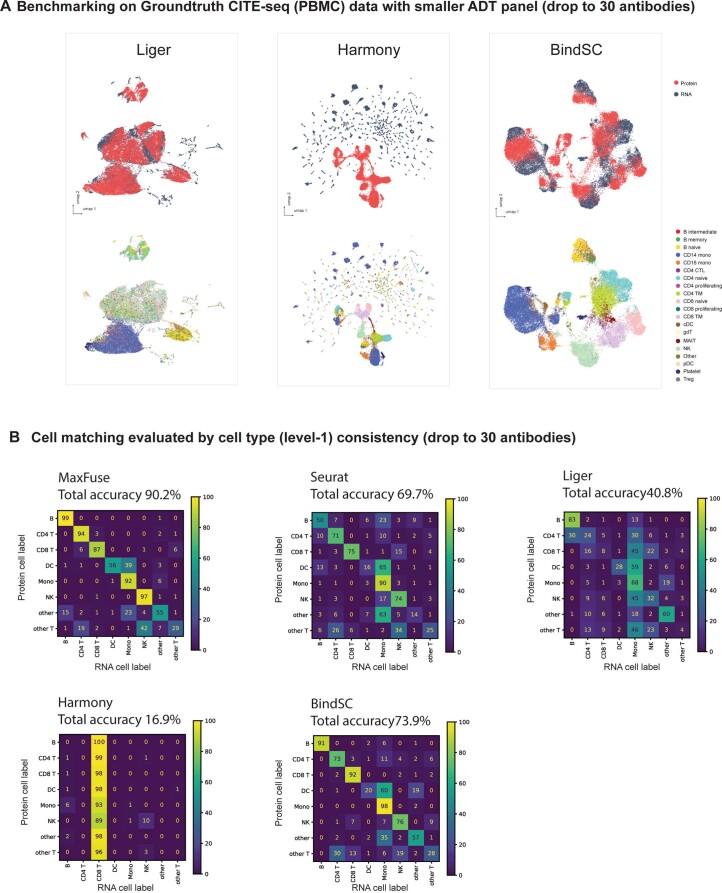
Benchmarking on ground-truth CITE-seq PBMC data with top 30 antibodies from Hao et al.^33^. **(A)** UMAP visualization of Liger, Harmony, and BindSC integration results, colored by modality (upper panel) or level 2 cell types (lower panel). **(B)** Cell matching accuracy results (cell type level 1) of different methods.

It is common to have an antibody panel that is of substantially smaller size than 228, especially for spatial proteomic datasets. To benchmark the performance of MaxFuse against existing methods with smaller antibody panels, we ordered the proteins according to their importance for differentiating cell types (see the Methods for details). We repeated the matching and integration process with the top 100, 50 and 30 most important proteins used in the matching and integration process. With each panel size, we ran the experiment over five independent repetitions with 10,000 randomly subsampled cells, and averaged the cell-type annotation matching accuracy (level 1 and level 2), FOSCTTM and FOSKNN scores across repetitions (Fig. 2c). Regardless of panel size, MaxFuse consistently outperformed other methods. Additionally, MaxFuse successfully mitigated the effect of reduced panel size on integration quality: even when the antibody panel size was reduced to 30, MaxFuse had approximately 90% accuracy for level 1 annotation, whereas accuracy of the other methods ranged from around 15% to 75% (Extended Data Fig. 2). With a reduced panel of 30 antibodies, the integrated UMAP embedding^38^ produced by other methods blurred the distinction between cell types, whereas MaxFuse embedding still accurately captured the subtle structure of highly granular cell subtypes, such as the B cell subpopulations (Fig. 2d and Extended Data Fig. 2).

**Extended Data Fig. 3:**
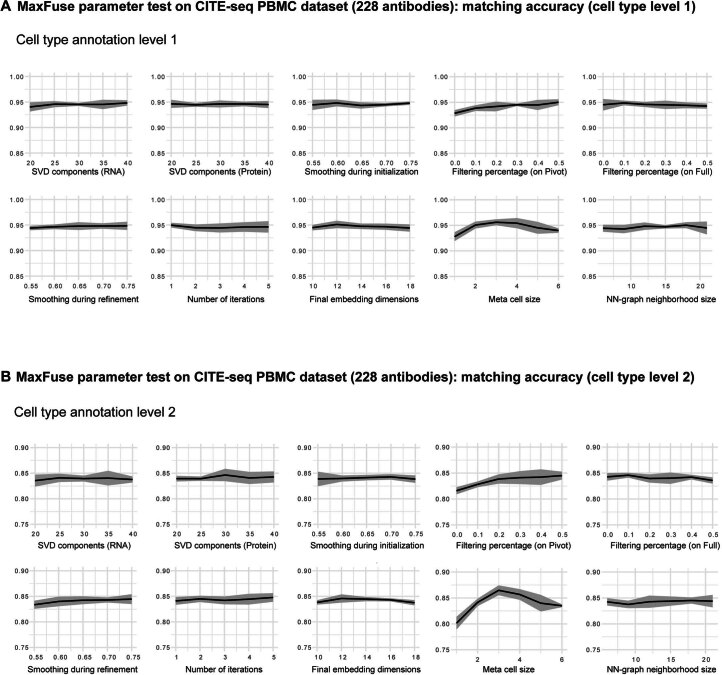
Benchmarking of robustness to tuning parameters in MaxFuse integration on CITE-seq PBMC with all 228 antibodies from Hao et al.^33^, evaluated by matching accuracy at two levels. **(A)** Matching accuracy (cell type level 1) versus a range of SVD components for different modalities, smoothing weights during initialization and refinement, filtering percentages on pivot and on full matching, number of iterations, final CCA embedding dimensions, meta-cell size, and NN-graph neighborhood size. Line indicates mean value and shadow indicates 95% CI on both sides. **(B)** Matching accuracy (cell type level 2) versus a range of SVD components for different modalities, smoothing weights during initialization and refinement, filtering percentages on pivot and on full matching, number of iterations, final CCA embedding dimensions, meta-cell size, and NN-graph neighborhood size. Line indicates mean value and shadow indicates 95% CI on both sides.

**Extended Data Fig. 4:**
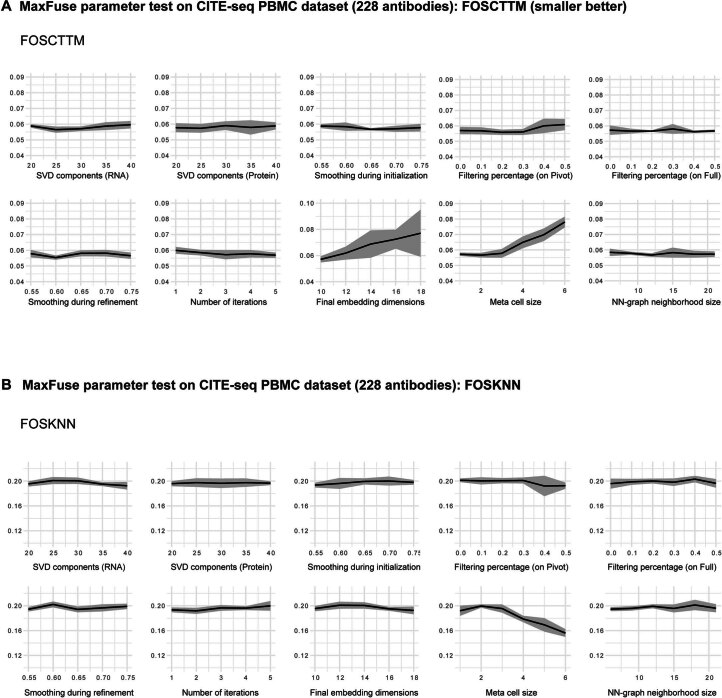
Benchmarking of robustness to tuning parameters in MaxFuse integration on CITE-seq PBMC with all 228 antibodies from Hao et al.^33^, evaluated by FOSCTTM and FOSKNN. **(A)** FOSCTTM scores versus a range of SVD components for different modalities, smoothing weights during initialization and refinement, filtering percentages on pivot and on full matching, number of iterations, final CCA embedding dimensions, meta-cell size, and NN-graph neighborhood size. Line indicates mean value and shadow indicates 95% CI on both sides. **(B)** FOSKNN scores versus a range of SVD components for different modalities, smoothing weights during initialization and refinement, filtering percentages on pivot and on full matching, number of iterations, final CCA embedding dimensions, meta-cell size, and NN-graph neighborhood size. Line indicates mean value and shadow indicates 95% CI on both sides.

In addition, we evaluated the impact of tuning parameter choice on MaxFuse integration results using ground-truth CITE-seq PBMC data. The investigated tuning parameters include matrix singular value decomposition components used for different modalities, smoothing weights used during initialization and refinement, number of refinement iterations, dimension for final canonical correlation analysis (CCA) embedding, filtering percentages on pivot and on full matching, meta-cell size and nearest-neighbor graph neighborhood size. Benchmarking on both the full panel of 228 antibodies and a reduced panel of the 50 most informative antibodies revealed that MaxFuse performance was robust with respect to the investigated tuning parameters (Extended Data Figs. 3 and 4 and Supplementary Figs. 1 and 2). Furthermore, we assessed the performance of MaxFuse when certain cell subpopulations were absent from one modality. Benchmark tests considering three different missing cell subpopulations in protein modality showed that MaxFuse was robust with respect to mismatch of cell populations between the two modalities (Supplementary Table 5).

### Benchmarking on multiple ground-truth multiome modalities

We further benchmarked MaxFuse on four additional single-cell multiome datasets. The first was a CITE-seq dataset of human bone marrow mononuclear cells that provides cell-matched measurements of the full transcriptome along with an antibody panel of size 25 (ref. ^33^). The second was an Ab-seq dataset, also of bone marrow mononuclear cells, with an antibody panel of size 97 and the whole transcriptome^39^. The third was an ATAC with select antigen profiling sequencing (ASAP-seq) PBMC dataset^40^ with 227 antibodies and the whole epigenome measured in ATAC fragments. The fourth was a transcription, epitopes, and accessibility sequencing (TEA-seq) PBMC dataset^41^ where we focused on the simultaneous measurements of 46 antibodies and the whole epigenome measured in ATAC fragments. Together, these datasets represent a diverse collection of measurement technologies over different modality pairs. We benchmarked the performance of MaxFuse against Seurat (V3), Liger, Harmony and BindSC on these datasets. For datasets with simultaneous RNA and protein features, we linked each protein to its coding gene. For datasets with simultaneous ATAC and protein measurements, we linked each protein to the gene activity score^42^ computed from the ATAC fragments mapping near its coding gene. The known cell–cell correspondences across modalities were masked in the integration stage for all methods, but used afterwards for evaluation.

**Fig. 3:**
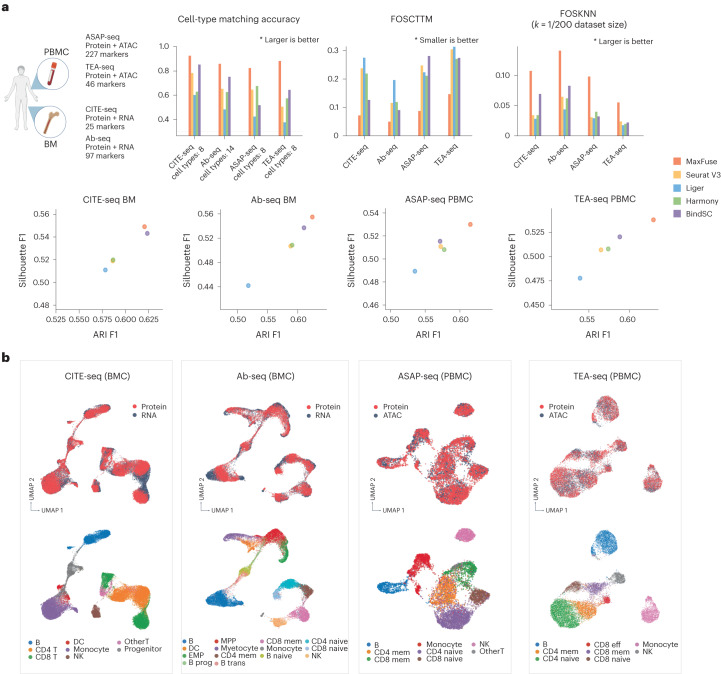
Benchmarking of MaxFuse versus other integration methods across multiple ground-truth data types. **a**, Four different multiome datasets, generated by different technologies, were benchmarked. Cell-type matching accuracy, FOSCTTM, FOSKNN (with *k* = 0.5% total cell counts of each dataset), and ARI and Silhouette F1 were evaluated across all five methods. **b**, UMAP visualization of MaxFuse integration results for the four ground-truth multiome datasets, colored by modality (top panel) and cell type (bottom panel). BM, bone marrow; DC, dendritic cells; EMP, erythro-myeloid progenitors; mem, memory; prog, progenitor; trans, transitional.

We compared the performances of MaxFuse and the other four methods on these datasets based on cell-type annotation matching accuracy, FOSCTTM, FOSKNN (*k* set as 1/200 dataset size), Silhouette F1 score and Adjusted Random Index (ARI) F1 score. Overall, MaxFuse outperformed other methods, often by a sizable margin (Fig. 3a and Supplementary Figs. 3–6). UMAPs of MaxFuse cross-modal joint embeddings for each dataset are shown in Fig. 3b. Across the integration scenarios, MaxFuse mixed different modalities well in joint embeddings while retaining separation between cell types. Compared with UMAPs of joint embeddings produced by other methods, MaxFuse consistently achieves substantial improvements (Fig. 3b and Supplementary Figs. 3–6).

**Extended Data Fig. 5:**
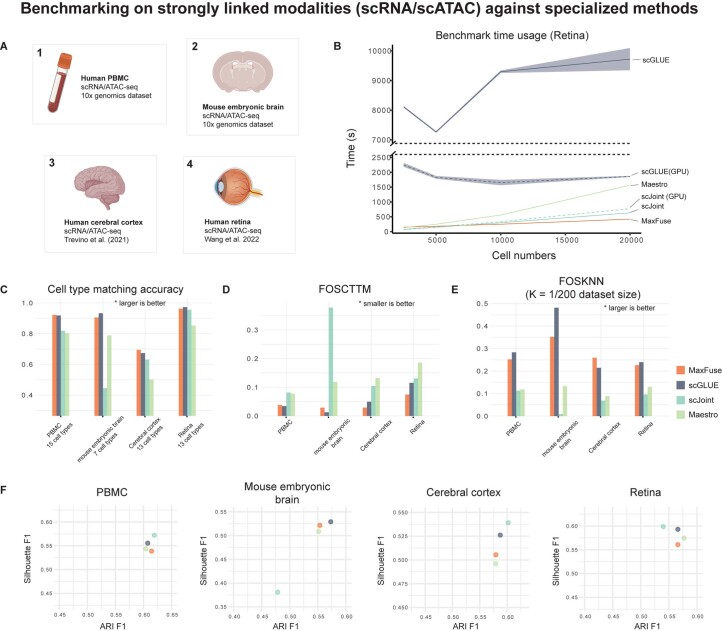
Benchmarking of MaxFuse on ground-truth strongly linked modalities against modality specialized methods. **(A)** Four ground-truth scRNA/scATAC multiome datasets used for the benchmarking of MaxFuse against specialized methods for scRNA/scATAC integration tasks (scGLUE, scJoint and Maestro). **(B)** Runtime benchmark on Retina data^48^ with different subsample sizes (2,500, 5,000, 10,000, and 20,000 cells). Methods with GPU option (scJoint and scGLUE) were tested under both CPU-only and GPU modes. MaxFuse, scJoint (CPU-only), and scGLUE (CPU-only) experiments were performed on a MacBook Pro with M1 Max CPU. scJoint (GPU) and scGLUE (GPU) experiments were performed on a Linux workstation with dual Intel i9-10980XE CPUs and dual NVIDIA Quadro RTX 8000 GPUs. Due to M1 silicon incompatibility, Maestro experiments were performed on a Linux workstation with dual Intel i9-10980XE CPUs and dual NVIDIA Quadro RTX 8000 GPUs. The reported Maestro runtimes were calibrated against scJoint runtimes on both computing platforms to ensure fair comparison. Line indicates mean value and shadow indicates 95% CI on both sides. **(C)** Cell matching accuracy (cell type level) of different methods on the four datasets. **(D)** FOSCTTM scores of different methods on the four datasets. **(E)** FOSKNN scores of different methods on the four datasets. **(F)** Silhouette F1 scores (y axis) and ARI F1 scores (x axis) of joint embeddings produced by different methods on the four datasets.

We also considered integration of scRNA-seq and scATAC-seq data. This is a representative example of integrating strongly linked modalities for which multiple methods have demonstrated feasibility^18,19,22^. It has been shown in ref. ^43^ that, in terms of cell population structure, the information shared across RNA and ATAC is much higher than the information shared between RNA and protein for commonly used targeted protein panels. Thus, RNA and ATAC data have stronger linkage and should be easier to integrate. We benchmarked MaxFuse against state-of-the-art methods (Maestro^44^, scJoint^45^ and scGLUE^19^) that are specific for RNA–ATAC integration on four public multiome datasets that simultaneously measured the chromatin accessibility and transcriptome expression for each cell: cells from human PBMCs^46^, cells from embryonic mouse brain at day 18 postconception^46^, cells from developing human cerebral cortex^47^ and cells from human retina^48^ (Extended Data Fig. 5a). The integration quality criteria described in the previous subsection were used to assess all methods. MaxFuse achieved best or close-to-best integration performance among the tested methods, and was comparable to scGLUE (Extended Data Fig. 5c–f). However, MaxFuse is computationally much faster than scGLUE. For example, for the integration of a dataset of 20,000 cells, MaxFuse completed within 5 min on a MacBook Pro laptop with M1 Max CPU, while scGLUE took hours to complete the job on the same platform. Even with CUDA GPU acceleration, scGLUE still used around 30 min to finish on a computing platform with dual Intel i9-10980XE CPUs and dual NVIDIA Quadro RTX 8000 GPUs (Extended Data Fig. 5b).

### MaxFuse enables information-rich spatial pattern discovery

**Fig. 4:**
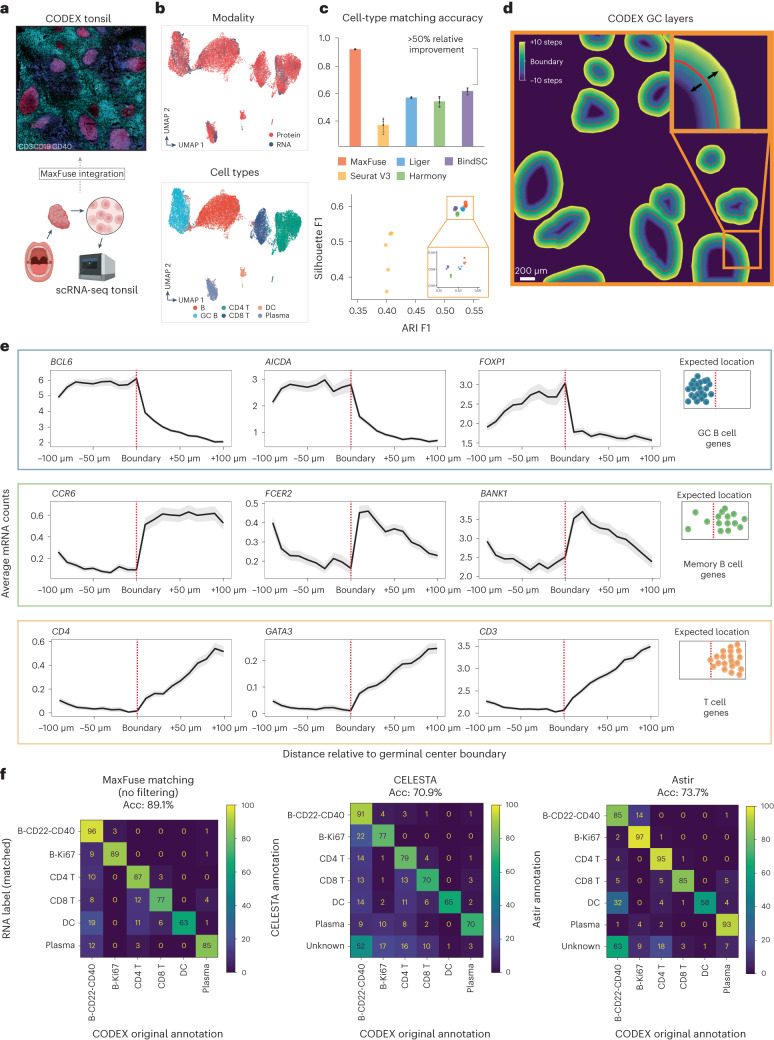
MaxFuse enables information-rich spatial pattern discovery. **a**, Schematic of integration of CODEX data from Kennedy-Darling et al.^49^ (upper panel), with scRNA-seq data from King et al.^50^ (lower panel) obtained from human. **b**, UMAP visualization of MaxFuse integration of tonsil CODEX and scRNA-seq data, colored by modality (upper panel) and cell type (lower panel). **c**, Metrics (cell-type matching accuracy, Silhouette F1 score and ARI F1 score) evaluating performance for MaxFuse and other methods. Five batches of CODEX and scRNA-seq cells (10,000 scRNA-seq cells and 30,000 CODEX cells in each batch) were randomly sampled and used for benchmarking for all methods. The barplot of cell-type matching accuracy shows mean value with 95% CI for each method, with raw values from five random samples plotted as dots. **d**, Illustration of cell layers extending inwards/outwards from the GC boundary. Each layer consisted of 30 pixels (~11 μm). A total of ten layers extending in each direction were examined. **e**, Average messenger RNA counts (linked by MaxFuse) across cells in each layer plotted versus the position of the layer in reference to the GC boundary (inward on the left of boundary, outward on the right). Expected expression profiles relative to the GC boundary are shown to the right of each group of three transcripts. Each line indicates mean value with the shadow area covering 95% CI for the mean at each position. Except for *CD3* and *CD4*, none of the other seven reported transcripts had its corresponding protein measured in the CODEX panel. **f**, Benchmarking of MaxFuse and other methods for cell-type annotation on human tonsil CODEX data^49,50^. Automated annotations were compared with human-expert annotations of human tonsil CODEX data. Left, MaxFuse cell-type annotation of CODEX cells by label transfer of matched human tonsil scRNA-seq cells. Middle, CELESTA^57^ cell-type annotation by using CODEX protein expression levels and previous knowledge on marker expression and cell population information. Right, Astir^58^ cell-type annotation by using CODEX protein expression levels and previous knowledge on marker expression and cell population information. Acc, accuracy; DC, dendritic cells.

MaxFuse is motivated by scenarios where the signal-to-noise ratio in the cross-modal linked features is low. Weak linkages are especially common in spatial-omic data types due to technical limitations. For example, high-resolution spatial proteomic methods such as CODEX, MIBI-TOF, IMC and CosMx SMI can profile, at subcellular resolution, a panel of 30–100 proteins^10–13^. Integration of such spatial proteomics datasets with single-cell transcriptomic and epigenomic datasets of the same tissue is often of interest, but is particularly challenging due to the small number of markers in the spatial dataset and the weak linkage between modalities which is caused by both biological and technical differences. To test MaxFuse on this type of cross-modal integration, we evaluated its performance on integrating a CODEX multiplex imaging dataset obtained using 46 markers^49^ with scRNA-seq data^50^ of human tonsils from two separate studies (Fig. 4a). MaxFuse produced an embedding that integrated the two modalities while preserving the cell population structure (Fig. 4b).

**Extended Data Fig. 6:**
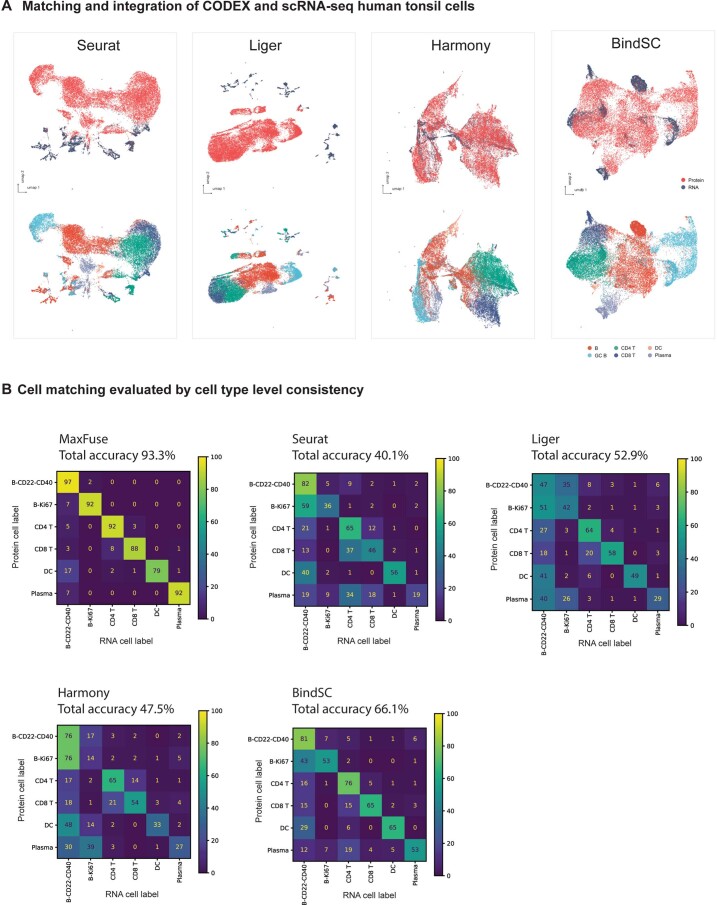
Benchmarking on human tonsil CODEX and scRNA-seq data from Kennedy-Darling et al.^49^ and King et al.^50^. **(A)** UMAP visualization of Seurat, Liger, Harmony, and BindSC integration results, colored by modality (upper panel) or cell types (lower panel). **(B)** Cell matching accuracy results (cell type level) of different methods.

Based on the predescribed benchmarking metrics, MaxFuse is the only method capable of integrating spatial proteomic and scRNA-seq data. Seurat (V3), Liger, BindSC and Harmony failed to produce an embedding that integrates the two modalities while preserving the cell population structure (Fig. 4b and Extended Data Fig. 6). Evaluation results based on cell-type matching accuracy are consistent with evaluation results based on the joint embedding. At the level of the six major cell types presented in the tissue, MaxFuse achieved high label transfer accuracy (93.3%), while the other methods failed to preserve cell-type distinctions (40–60%; Fig. 4b and Extended Data Fig. 6).

**Extended Data Fig. 7:**
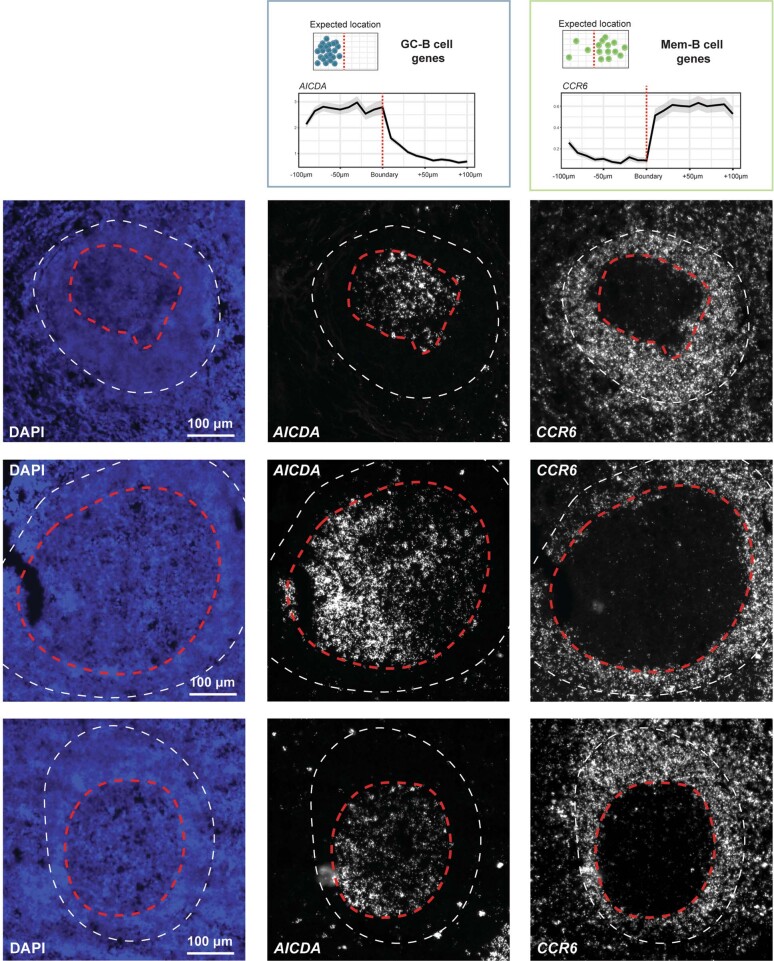
Experimental validation of MaxFuse predicted mRNA spatial expression pattern. RNAscope was performed on fresh-frozen human tonsil tissue to validate the spatial expression pattern of *AICDA* and *CCR6*. The top row demonstrates MaxFuse predicted mRNA spatial expression patterns of *AICDA* and *CCR6* (taken from Fig. 4E). Three representative germinal centers were shown in the second to the fourth row, with the red line indicating germinal center boundary and the white line indicating *CCR6* boundary. Within each row, from left to right: nucleus (DAPI) channel, *AICDA* RNAscope channel, and *CCR6* RNAscope channel. Only 3 representative GCs were shown in the figure due to the limitation of space. The conclusion was made by validating > 20 individual GCs.

To assess whether MaxFuse preserves subtle spatial variations within a cell type that are captured by CODEX, we manually delineated the boundaries of each individual germinal center (GC) from the CODEX tonsil images based on CD19, CD21 and Ki67 protein expression patterns. We then extended outward or inward from these boundaries, with each step covering roughly one layer of cells (one step = 30 pixels erosion/dilation) (Fig. 4c). For each layer of cells, we calculated the average counts of specific genes, based on the scRNA-seq cells matched to CODEX cells in that layer. We then asked if known position-specific gene expression patterns relative to the GC boundary are recovered in the integrated scRNA-seq data. Indeed, MaxFuse was able to reconstruct the spatial pattern of the GC from disassociated transcriptomic data (Fig. 4d,e): for GC-specific transcripts *BCL6*, *AICDA* and *FOXP1* (refs. ^51–53^) which relate to GC functionality, we observed high expression within the boundary and a sharp drop in expression after passing the boundary layer; for transcripts related to B cell memory, *CCR6*, *BANK1* and *FCER2* (refs. ^53–55^), which should be enriched in B cells exiting from the GC, we indeed saw a gradual increase outside of the GC and then a quick decrease as the layer fully expanded into the T cell region; and finally for T cell-related transcripts, for example *CD4*, *GATA3* and *CD3* (ref. ^56^), we indeed saw a rapid increase outside of the GC boundary but no expression within. In comparison, the integration produced by other methods did not accurately reconstruct the GC spatial pattern (Supplementary Fig. 7). Except for *CD3* and *CD4*, none of the other seven transcripts had its corresponding protein measured in the CODEX panel. We also followed with experimental validation via RNAscope, where we observed consistent spatial patterns of *AICDA* and *CCR6* in human tonsil, as predicted by MaxFuse integration (Extended Data Fig. 7).

Furthermore, MaxFuse can be utilized for automated cell-type annotation of CODEX cells, given that the scRNA-seq data to be matched are annotated. We evaluated the automated annotations on all CODEX cells produced by MaxFuse, comparing them with those generated by two cutting-edge CODEX cell-type annotation methods, CELESTA^57^ and Astir^58^. This comparison was benchmarked against annotations made by human experts. MaxFuse achieved an annotation accuracy of nearly 90%, substantially improving upon these two methods for direct annotation of CODEX data, which had accuracy within the 70–75% range (Fig. 4f).

### Tri-modal atlas-level integration with MaxFuse

**Fig. 5:**
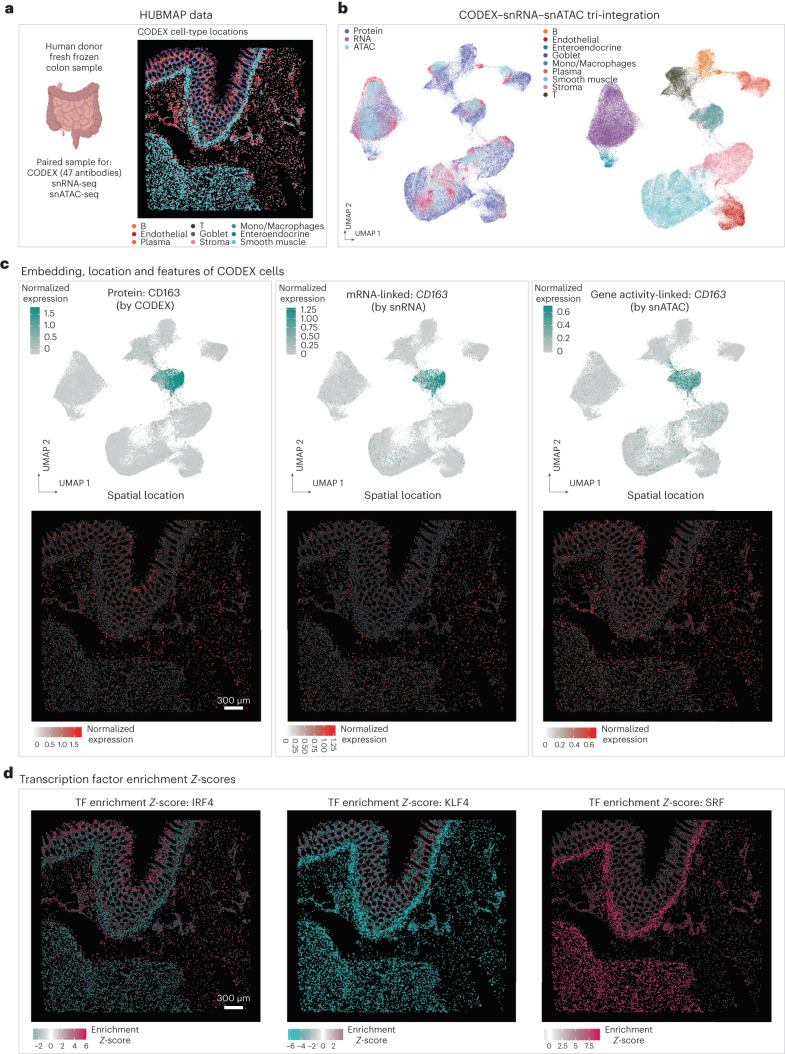
MaxFuse enables tri-modal integration with HUBMAP data. **a**, Overview of CODEX, snRNA-seq and snATAC-seq single-cell human intestine data from the HUBMAP consortium (left). Representative cell-type locations based on CODEX data (right). Colon and small bowel data were integrated by MaxFuse, respectively, and this figure shows part of the colon data (CODEX data from one donor; snRNA-seq and snATAC-seq data from four donors). **b**, UMAP visualization of the tri-modal integration embedding produced by MaxFuse, colored by modality: protein, RNA and ATAC (left panel) and colored by cell type (right panel). **c**, Upper row, UMAP visualization of CODEX cells based on the integration embedding, overlaid with CD163 protein expression (from CODEX cells themselves, left panel), *CD163* mRNA expression (from matched snRNA-seq cells, middle panel) and *CD163* gene activity score (from matched snATAC-seq cells, right panel). Lower row, spatial locations of CODEX cells based on *x*–*y* positions of centroids, overlaid with the same expression features as in the corresponding panels of the upper row. **d**, Spatial locations of CODEX cells based on *x*–*y* positions of centroids, overlaid with the transcription factor motif enrichment scores (*Z*-scores, calculated by chromVAR^60^), based on their matched snATAC-seq cells. TF, transcription factor.

**Extended Data Fig. 8:**
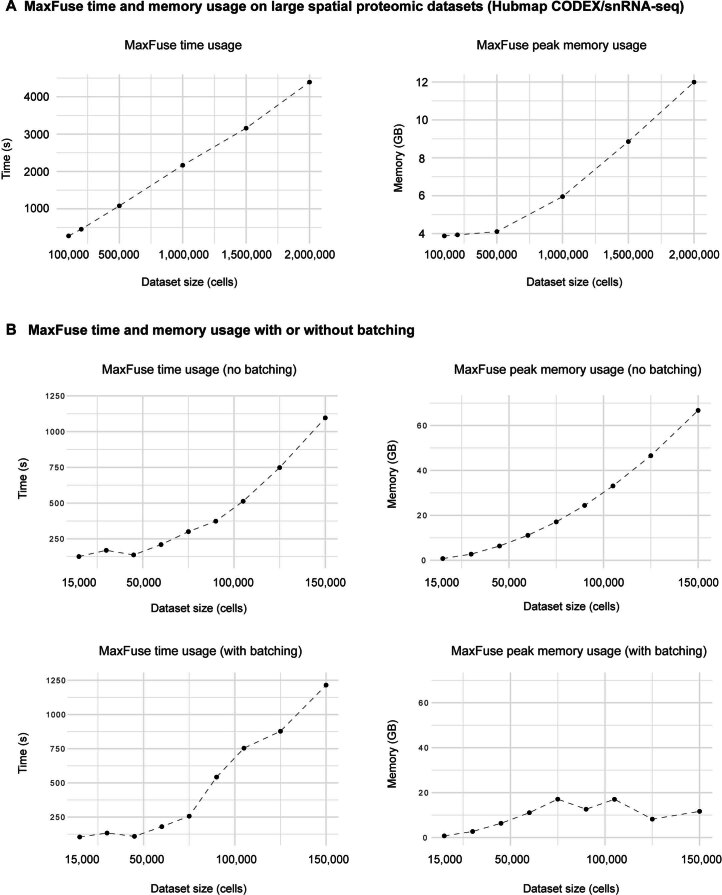
Benchmarking of MaxFuse run time and memory usage on integrating large datasets. **(A)** Run time and peak memory usage of MaxFuse on large spatial proteomic-related integration (with batching): HUBMAP colon CODEX & snRNA-seq^31^ integration where up to 2 million CODEX cells were tested. In all settings, around 18,000 snRNA-seq cells were used. **(B)** Run time and peak memory usage of MaxFuse without batching (top row) and with batching (bottom row), tested on randomly sampled subsets of different sizes from the CITE-seq PBMC data^33^.

In the consortium-level effort to generate a comprehensive atlas across different regions of the human intestine, colon and small bowel tissues from healthy human donors were collected and systematically profiled by CODEX, snRNA-seq and snATAC-seq^31^. We applied MaxFuse to the integration of these three datasets obtained from analyses of colon (Fig. 5a), with the goal of constructing high-resolution spatial maps of full transcriptome RNA expression and transcription factor binding accessibility. We first conducted pairwise alignment of cells between protein (CODEX) and RNA (snRNA-seq), and cells between RNA (snRNA-seq) and ATAC (snATAC-seq), as previously described. The two sets of bimodal cell-pairing pivots were then ‘chained’ together, with the pivot cells in the RNA modality serving as the intermediary. This ‘chaining’ created a set of pivots linking all three modalities: protein, RNA and ATAC. Subsequently, we used these pivots to calculate a tri-omic embedding via generalized CCA (gCCA)^21,59^. This allowed calculation of a joint embedding of the three modalities (Fig. 5b). The MaxFuse integration preserved distinctions between major cell types, and modalities were mixed within each cell type. See Supplementary Fig. 8 for a comparison between using RNA and using ATAC as the baseline (intermediary) modality. Additionally, the design of batching in MaxFuse allowed the integration of atlas-level datasets with limited time and space resources (Extended Data Fig. 8).

**Extended Data Fig. 9:**
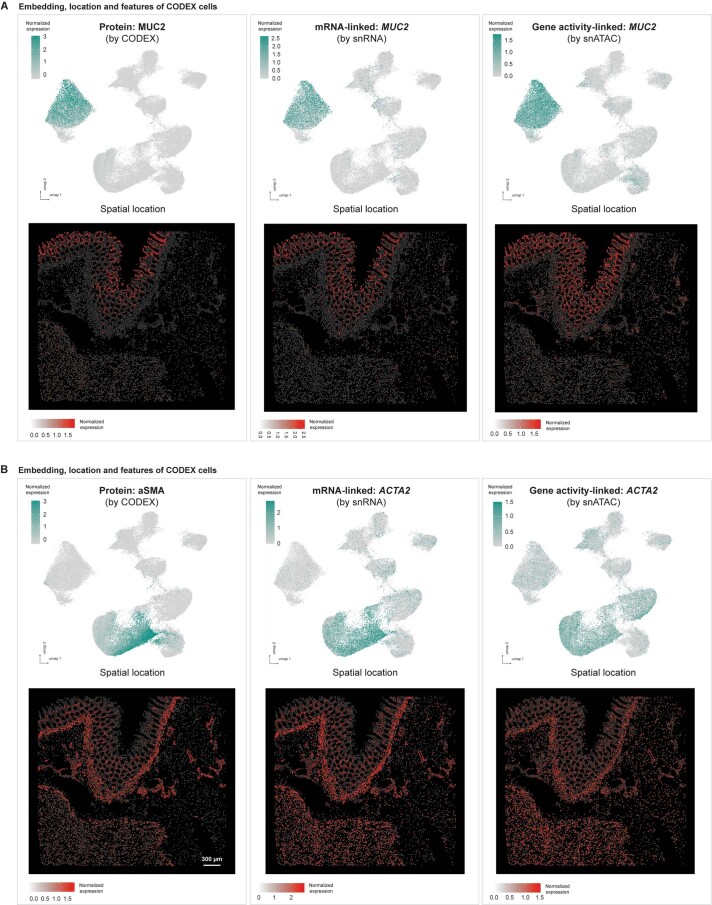
Additional markers showing consistent expression patterns across tri-modalities for Fig. 5. **(A)** Upper row: UMAP visualization of CODEX cells based on the integration embedding, overlaid with MUC2 protein expression (from CODEX cells themselves, left panel), *MUC2* RNA expression (from matched snRNA-seq cells, middle panel), *MUC2* gene activity score (from matched snATAC-seq cells, right panel). Lower row: Spatial locations of CODEX cells based on their centroids’ x-y positions, overlaid with the same expression features as in the corresponding panels of the upper row. **(B)** Upper row: UMAP visualization of CODEX cells based on the integration embedding, overlaid with aSMA protein expression (from CODEX cells themselves, left panel), *ACTA2* RNA expression (from matched snRNA-seq cells, middle panel), *ACTA2* gene activity score (from matched snATAC-seq cells, right panel). Lower row: Spatial locations of CODEX cells based on their centroids’ x-y positions, overlaid with the same expression features as in the corresponding panels of the upper row.

Effectively, the MaxFuse integration produced a joint profile of protein abundance, RNA expression and chromatin accessibility at single-cell spatial resolution on the same tissue section. To confirm the validity of this tri-modal integration, we inspected whether CODEX’s protein abundance aligned spatially with the expression and chromatin activity of the protein-coding gene, the spatial measurements of the latter two modalities imputed based on the MaxFuse integration. In one example, the protein expression, RNA expression and gene activity of CD163 were, as expected for this macrophage marker, uniquely enriched in the macrophage cell cluster (Fig. 5c, top row). Furthermore, protein, RNA and ATAC activities of this gene all localized to the same spatial positions on the tissue section (Fig. 5c, bottom row). See Extended Data Fig. 9 for additional examples.

**Extended Data Fig. 10:**
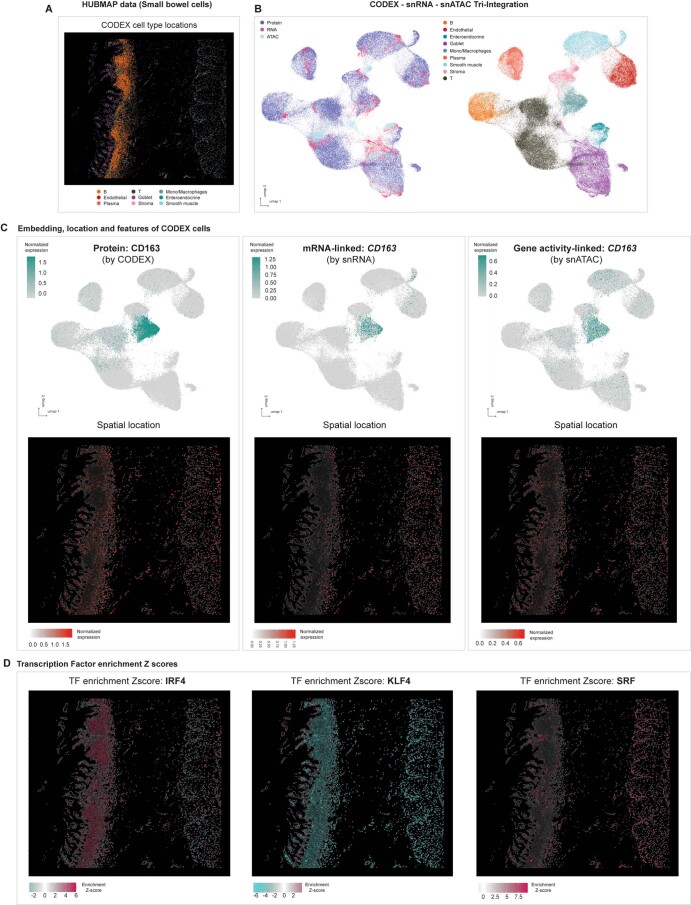
Tri-modal integration with MaxFuse on HUBMAP small bowel data. **(A)** Representative cell type spatial locations on CODEX HUBMAP small bowel tissue. **(B)** UMAP visualization of the tri-modal integration embedding produced by MaxFuse, colored by modality: Protein, RNA and ATAC (left panel) and colored by cell type (right panel). **(C)** Upper row: UMAP visualization of CODEX cells based on the integration embedding, overlaid with CD163 protein expression (from CODEX cells themselves, left panel), *CD163* RNA expression (from matched snRNA-seq cells, middle panel), *CD163* gene activity score (from matched snATAC-seq cells, right panel). Lower row: Spatial locations of CODEX cells based on their centroids’ x-y positions, overlaid with the same expression features as in the corresponding panels of the upper row. **(D)** Spatial locations of CODEX cells based on their centroids’ x-y positions, overlaid with the transcription factor motif enrichment scores (Z-scores, calculated by chromVAR^60^), based on their matched snATAC-seq cells.

With the integration of the snATAC-seq and CODEX data, we were able to map the spatial enrichment of transcription factor binding site accessibility. For each transcription factor, we first computed a motif enrichment score for each cell in the snATAC-seq data using chromVAR^60^, and then the scores were transferred to the CODEX spatial positions based on the MaxFuse integration. Figure 5d shows such spatial profiles for three transcription factors. Binding motifs of *IRF4*, a key regulator in immune cell differentiation^61^, had increased accessibility in the immune-enriched compartments of the mucosa and submucosa layers^31^. Binding motifs of *KLF4*, known to be required for the terminal differentiation of goblet cells^62^, had heightened accessibility in the colonic crypts of the mucosa layer where goblet cells mature. Finally, binding motifs of *SRF*, a master regulator of smooth muscle gene expression^63^, had heightened accessibility in neighborhoods that are enriched for smooth muscle cells. In addition, we performed the same analysis on the HUBMAP data collected on small bowel and MaxFuse showed consistent results (Extended Data Fig. 10).

### Additional benchmarking of MaxFuse

We further compared the integration quality within MaxFuse results, across different smoothing schemes (Supplementary Fig. 9), and between pivot and nonpivot cells (Supplementary Fig. 10 and Supplementary Table 1). We validated the improved gene imputation accuracy by MaxFuse-enabled matching in a ground-truth multiome dataset, using targeted proteomic features to predict transcript expression at single-cell level (Supplementary Fig. 11). One important potential application of MaxFuse is imputing unmeasured features (for example, transcripts) in spatial proteomic datasets. We benchmarked the effect on integration quality of sequentially reduced antibody panel sizes (Supplementary Fig. 12) and the area-level gene imputation correlation by artificially dropping protein features in CODEX data (Supplementary Fig. 13).

## Discussion

Most existing methods for cross-modal data integration were developed for integration across strongly linked modalities, and their performances decay significantly as the strength of cross-modal linkage weakens. MaxFuse is motivated by and focuses on the challenging case of weak linkage, which has become increasingly common as many emerging study designs include spatial data with targeted marker panels to be collected jointly with single-cell sequencing data.

MaxFuse relies on two key processes to overcome weak linkage. The first is a ‘fuzzy smoothing’ procedure that denoises the linked features by moving their values towards their graph-smoothed values, with the graph determined by all features. The second is an iterative refinement procedure that improves the cross-modal matching through iterative cycles of coembedding, graph-smoothing and matching. This ensures that information from all features, in both modalities, is used to generate the final matching. We demonstrated that MaxFuse substantially improves upon state-of-the-art methods, achieving accurate integration of data from targeted protein assays with data from transcriptome- and epigenome-level assays. The applicability of MaxFuse is general. For strong linkage scenarios, MaxFuse accuracy was comparable to scGLUE, a state-of-the-art method based on deep learning, but at a considerably lower computational cost. In addition, when joint embedding coordinates from other integration methods are available, these coordinates could serve as linked features in MaxFuse. The light computation architecture and the flexibility in incorporating domain knowledge and existing integration results make the MaxFuse framework applicable to a wide range of cross-modal integration tasks.

## Methods

### The MaxFuse pipeline

#### Input preparation

Consider a pair of datasets, $Y\in {{\mathbb{R}}}^{{N}_{y}\times {p}_{y}}$ and $Z\in {{\mathbb{R}}}^{{N}_{z}\times {p}_{z}}$, from two modalities (termed *Y*-modality and *Z*-modality for exposition convenience), with each row corresponding to a cell and each column a feature. In the ensuing discussion, we treat *Y* as the modality with a higher signal-to-noise ratio. For concreteness, one can think of *Y* as an snRNA-seq dataset and *Z* as a CODEX dataset. Suppose there are two known functions, ${f}_{y}:{{\mathbb{R}}}^{{p}_{y}}\to {{\mathbb{R}}}^{s}$ and ${f}_{z}:{{\mathbb{R}}}^{{p}_{z}}\to {{\mathbb{R}}}^{s}$, such that $f_y(\mathbf{y})$ predicts the values of $f_z(\mathbf{z})$ in a cell if the measured values under *Y*-modality are ${\mathbf{y}}$ in that cell and those under *Z*-modality are ${\mathbf{z}}$. For any matrix *A* with *p*_*y*_ columns, let *f*_*y*_(*A*) denote the matrix with *s* columns and the same number of rows as *A*, obtained from applying *f*_*y*_ on each row of *A* and stacking the outputs as row vectors. For any matrix *B* with *p*_*z*_ columns, *f*_*z*_(*B*) is analogously defined. We define ${Y}^{\circ }={f}_{y}(Y)\in {{\mathbb{R}}}^{{N}_{y}\times s}$ and ${Z}^{\circ }={f}_{z}(Z)\in {{\mathbb{R}}}^{{N}_{Z}\times s}$. In the snRNA-seq versus CODEX example, if one has a crude prediction for a subset *S* (with size $\left\vert S\right\vert =s$) of the proteins, then $f_z(\mathbf{z})={\mathbf{z}}_S$ returns the subvector indexed by *S* while ${f}_{y}({\bf{y}})={\hat{\mathbf{z}}}_{S}$ predicts the observed CODEX values for these proteins based on transcriptomic information of a cell. In summary, we start with a pair of original datasets (*Y*, *Z*) and a pair of datasets (${Y^{\circ}}$, ${Z^{\circ}}$), where the columns of the latter have one-to-one correspondence based on domain knowledge. The columns of ${Y^{\circ}}$ and ${Z^{\circ}}$ can be learned feature-wise prediction functions, as described above, or learned coembedding coordinates from some model trained on multi-omics data.

*Meta-cell construction*. To alleviate sparsity and to scale to large datasets, we start by constructing meta-cells. Let *n*_*y*_ be the desired number of meta-cells. We first construct a nearest-neighbor graph of the rows of *Y*, apply Leiden clustering with an appropriate resolution level to obtain *n*_*y*_ clusters and average over the rows within each cluster to obtain the features for each meta-cell. Consequently, we obtain ${Y}_{{\mathtt{m}}}\in {{\mathbb{R}}}^{{n}_{y}\times {p}_{y}}$. Using this clustering structure (induced by *Y*), we can average feature vectors in ${Y^{\circ}}$ to obtain ${Y}_{{\mathtt{m}}}^{\circ }\in {{\mathbb{R}}}^{{n}_{y}\times s}$. When desired, the same operation can be performed on the *Z*-modality to obtain ${Z}_{{\mathtt{m}}}\in {{\mathbb{R}}}^{{n}_{z}\times {p}_{z}}$ and ${Z}_{{\mathtt{m}}}^{\circ }\in {{\mathbb{R}}}^{{n}_{z}\times s}$. We recommend only constructing meta-cells for modalities that allow cell state differentiation at fine granularity. For example, if *Y*-modality contains snRNA-seq data and *Z*-modality contains CODEX data, then we would usually recommend to construct meta-cells only in *Y*-modality. The choices of meta-cell size for analyses reported in this work are given in Supplementary Table 2. In addition, in Extended Data Figs. 3 and 4 and Supplementary Figs. 1 and 2, we benchmarked robustness of results with respect to meta-cell size. Meta-cell sizes of 2–3 are optimal across the datasets we tested. After this curation step, we have two pairs of datasets, $({{Y}_{\mathtt{m}}},{{Z}_{\mathtt{m}}})$ and $({Y}_{{\mathtt{m}}}^{\circ },{Z}_{{\mathtt{m}}}^{\circ })$. The former pair can have completely distinct feature sets, while the latter pair must have matching feature sets with corresponding columns. In Fig. 1a, the former correspond to the pair of all-feature matrices, and the latter correspond to the pair of linked-feature matrices.

#### Fuzzy smoothing

Let ${G}_{Y}\in {\{0,1\}}^{{n}_{y}\times {n}_{y}}$ be a nearest-neighbor graph of ${Y}_{\mathtt{m}}$ where each row *i* is connected to ${k}_{i}^{Y}$ rows that are closest in a chosen similarity measure, including itself. So row *i* of *G*_*Y*_ has ${k}_{i}^{Y}$ entries equal to one and others zeros. In addition, all its diagonal entries are equal to one. Let ${{{{\mathcal{A}}}}}_{Y}({Y}_{{\mathtt{m}}})={K}_{Y}^{-1}{G}_{Y}{Y}_{{\mathtt{m}}}$ and ${{{{\mathcal{A}}}}}_{Y}({Y}_{{\mathtt{m}}}^{\circ })={K}_{Y}^{-1}{G}_{Y}{Y}_{{\mathtt{m}}}^{\circ }$ be locally averaged versions of ${Y}_{\mathtt{m}}$ and ${Y}_{{\mathtt{m}}}^{\circ }$ over *G*_*Y*_, respectively, where ${K}_{Y}={{{\rm{diag}}}}({k}_{1}^{Y},\ldots ,{k}_{{n}_{y}}^{Y})$. For a nearest-neighbor graph *G*_*Z*_, we define ${{{{\mathcal{A}}}}}_{Z}({Z}_{{\mathtt{m}}})$ and ${{{{\mathcal{A}}}}}_{Z}({Z}_{{\mathtt{m}}}^{\circ })$ in an analogous way. Finally, for any weight *w* ∈ [0, 1] and any matrices *A* and *B* with *n*_*y*_ and *n*_*z*_ rows, respectively, we define

1 $$\begin{array}{rcl}{{{{\mathcal{S}}}}}_{Y}(A;w)&=&wA+(1-w){{{{\mathcal{A}}}}}_{Y}(A),\\ {{{{\mathcal{S}}}}}_{Z}(B;w)&=&wB+(1-w){{{{\mathcal{A}}}}}_{Z}(B).\end{array}$$

In this way, we define ${\widetilde{Y}}_{{\mathtt{m}}}^{\circ }={{{{\mathcal{S}}}}}_{Y}({Y}_{{\mathtt{m}}}^{\circ };{w}_{0})$ and ${\widetilde{Z}}_{{\mathtt{m}}}^{\circ }={{{{\mathcal{S}}}}}_{Z}({Z}_{{\mathtt{m}}}^{\circ };{w}_{0})$ with *w*_0_ ∈ [0, 1]. In Fig. 1a, these are matrices with smoothed *Y*-modality linked features and smoothed *Z*-modality linked features, respectively. See Supplementary Table 3 for a list of smoothing weights used in data analyses reported in this work.

#### Initial matching via linear assignment

As the columns in ${\widetilde{Y}}_{{\mathtt{m}}}^{\circ }$ and in ${\widetilde{Z}}_{{\mathtt{m}}}^{\circ }$ have correspondences, we can compute an *n*_*y*_ × *n*_*z*_ distance matrix ${D^{\circ}}$ where ${D}_{ij}^{\circ }$ measures the distance between the *i*-th row in ${\widetilde{Y}}_{{\mathtt{m}}}^{\circ }$ and the *j*-th row in ${\widetilde{Z}}_{{\mathtt{m}}}^{\circ }$ after projecting to respective leading singular subspaces. We obtain an initial matching ${\widehat{\Pi }}^{\circ }$ as the solution to the linear assignment problem^32,64^:

2 $$\begin{array}{rcl}&\,{{\mbox{minimize}}}\,&\langle \Pi ,{D}^{\circ }\rangle \\ &\,{{\mbox{subject to}}}\,&\Pi \in {\{0,1\}}^{{n}_{y}\times {n}_{z}}\\ &&\mathop{\sum}\limits_{i}{\Pi }_{ij}\le 1,\forall j,\quad \mathop{\sum}\limits_{j}{\Pi }_{ij}\le 1,\forall i,\\ &&\mathop{\sum}\limits_{i,\,j}{\Pi }_{ij}={n}_{\min }.\end{array}$$

Here, ${n}_{\min }=\min \{{n}_{y},{n}_{z}\}$ and, for two matrices *A* and *B* of the same size, 〈*A*, *B*〉 = ∑_*i*,*j*_*A*_*ij*_*B*_*ij*_ denotes the trace inner product. The linear assignment problem in equation (2) can be efficiently solved by relaxing the first constraint to $\Pi \in {[0,1]}^{{n}_{y}\times {n}_{z}}$. The resulting linear program has the same solution as equation (2). The Python implementation we used is based on the shortest augmenting path approach summarized in ref. ^65^. The estimator ${\widehat{\Pi }}^{\circ }$ provides a relatively crude matching using only the information provided by the previous knowledge encapsulated in *f*_*y*_ and *f*_*z*_ which link features in the two modalities. By definition, ${\widehat{\Pi }}^{\circ }$ gives ${n}_{\min }$ pairs of matched rows between the two modalities, which we call initial pivots.

#### Cross-modality joint embedding and iterative refinement

*From matched pairs to joint embedding*. An estimated matching $\widehat{\Pi }$ induces a cross-modality joint embedding of ${Y}_{\mathtt{m}}$ and ${Z}_{\mathtt{m}}$. Let ${Y}_{{\mathtt{m}}}^{\,{\mathtt{r}}}\in {{\mathbb{R}}}^{{n}_{y}\times {r}_{y}}$ and ${Z}_{{\mathtt{m}}}^{\,{\mathtt{r}}}\in {{\mathbb{R}}}^{{n}_{z}\times {r}_{z}}$ collect the leading principal components of all features (that is, ${Y}_{\mathtt{m}}$ and ${Z}_{\mathtt{m}}$) in the two modalities, respectively. Here, the numbers of principal components to retain, that is, *r*_*y*_ and *r*_*z*_, are chosen based on data. For any matrix *A*, let [*A*]_*i*⋅_ denote its *i*-th row. Suppose $\{({i}_{\ell },{i}_{\ell }^{{\prime} }):\ell =1,\ldots ,{n}_{\min }\}$ are the matched pairs specified by $\widehat{\Pi }$. We perform CCA on data pairs

$$\left\{\left({\left[{Y}_{{\mathtt{m}}}^{\,{\mathtt{r}}}\right]}_{{i}_{\ell }\cdot },{\left[{Z}_{{\mathtt{m}}}^{\,{\mathtt{r}}}\right]}_{{i}_{\ell }^{{\prime} }\cdot }\right):\ell =1,\ldots ,{n}_{\min }\right\}$$

to obtain the leading $${r}_{\mathtt{cc}}$$ loading vectors for either modality, collected as the columns of $${\widehat{C}}_{y}={\widehat{C}}_{y}(\widehat{\Pi })$$ and $${\widehat{C}}_{z}={\widehat{C}}_{z}(\widehat{\Pi })$$, respectively. The joint embedding induced by $$\widehat{\Pi }$$ is then $${Y}_{{\mathtt{m}}}^{{\mathtt{cc}}}={Y}_{{\mathtt{m}}}^{\,{\mathtt{r}}}{\widehat{C}}_{y}\in {{\mathbb{R}}}^{{n}_{y}\times {r}_{{\mathtt{cc}}}}$$ and $${Z}_{{\mathtt{m}}}^{{\mathtt{cc}}}=$$$${Z}_{{\mathtt{m}}}^{{\mathtt{r}}}{\widehat{C}}_{z}\in {{\mathbb{R}}}^{{n}_{z}\times {r}_{{\mathtt{cc}}}}$$, the predicted canonical correlation (CC) scores of $${Y}_{{\mathtt{m}}}^{{\mathtt{r}}}$$ and $${Z}_{{\mathtt{m}}}^{{\mathtt{r}}}$$, respectively.

*Iterative refinement*. Let ${\widehat{\Pi }}^{(0)}={\widehat{\Pi }}^{\circ }$ be the initial matching obtained from equation (2). We fix a weight *w*_1_ ∈ [0, 1] and the embedding dimension ${r^{\mathtt{cc}}}$, and we refine the estimated matching by iterating the following steps for *t* = 1, …, *T*:

1. Compute joint embedding $\{{Y}_{{\mathtt{m}}}^{\,{\mathtt{cc}},(t)},{Z}_{{\mathtt{m}}}^{\,{\mathtt{cc}},(t)}\}$ induced by ${\widehat{\Pi }}^{(t-1)}$;
2. Apply fuzzy smoothing on joint embedding: ${\widetilde{Y}}_{{\mathtt{m}}}^{\,{\mathtt{cc}},(t)}={{{{\mathcal{S}}}}}_{Y}({Y}_{{\mathtt{m}}}^{\,{\mathtt{cc}},(t)},{w}_{1})$, ${\widetilde{Z}}_{{\mathtt{m}}}^{\,{\mathtt{cc}},(t)}={{{{\mathcal{S}}}}}_{Z}({Z}_{{\mathtt{m}}}^{\,{\mathtt{cc}},(t)},{w}_{1})$;
3. Calculate a distance matrix ${D}^{(t)}\in {{\mathbb{R}}}^{{n}_{y}\times {n}_{z}}$ where ${D}_{ij}^{(t)}$ measures the distance between ${[{\widetilde{Y}}_{{\mathtt{m}}}^{{\mathtt{cc}},(t)}]}_{i\cdot }$ and ${[{\widetilde{Z}}_{{\mathtt{m}}}^{{\mathtt{cc}},(t)}]}_{j\cdot }$, and obtain a refined matching ${\widehat{\Pi }}^{(t)}$ by solving equation (2) in which ${D^{\circ}}$ is replaced with *D*^(*t*)^.

Figure 1b illustrates the foregoing refinement iteration.

#### Propagation of matching and postprocessing

For downstream analyses, one would often like to find for each cell in *Y* a match in *Z*, or vice versa, and sometimes both ways. In addition, one often wants joint embedding of cells across different modalities in a common space. We now describe how MaxFuse achieves these goals.

*Filtering and final joint embedding*. Upon obtaining the matched pairs $\{({i}_{\ell },{i}_{\ell }^{{\prime} }):\ell =1,\ldots ,{n}_{\min }\}$ in ${\widehat{\Pi }}^{(T)}$, we rank them in descending order of ${D}_{{i}_{\ell }{i}_{\ell }^{{\prime} }}^{(T)}$ and only retain the top 100 × (1 − *α*)% pairs, where *α* is a user-specified filtering proportion (with a default *α* = 0). The retained pairs are called refined pivots. Then, we fit a CCA using the refined pivots and the corresponding rows in ${Y}_{\mathtt{m}}$ and ${Z}_{\mathtt{m}}$ to get the associated CCA loading matrices ${\widehat{C}}_{y}^{{\mathtt{e}}}\in {{\mathbb{R}}}^{{p}_{y}\times {r}^{{\mathtt{e}}}}$ and ${\widehat{C}}_{z}^{{\mathtt{e}}}\in {{\mathbb{R}}}^{{p}_{z}\times {r}^{{\mathtt{e}}}}$. Here the positive integer ${r^{\mathtt{e}}}$ is a user-specified dimension for final joint embedding. Finally, the joint embedding of the full datasets is given by ${Y}^{{\mathtt{e}}}=Y{\widehat{C}}_{y}^{{\mathtt{e}}}\in {{\mathbb{R}}}^{{N}_{y}\times {r}^{{\mathtt{e}}}}$ and ${Z}^{{\mathtt{e}}}=Z{\widehat{C}}_{z}^{{\mathtt{e}}}\in {{\mathbb{R}}}^{{N}_{z}\times {r}^{{\mathtt{e}}}}$, respectively. In Fig. 1c, they correspond to the *Y*-modality embedding and *Z*-modality embedding matrices.

*Using pivots to propagate matching*. For each row index *i* ∈ {1, …, *n*_*y*_} in *Y*-modality that does not have a match in *Z*-modality, MaxFuse searches for the nearest neighbor of the *i*-th row in ${\widetilde{Y}}_{{\mathtt{m}}}={{{{\mathcal{S}}}}}_{Y}({Y}_{{\mathtt{m}}};{w}_{0})$ that belongs to some refined pivot. Suppose the nearest neighbor is the *j*_*i*_-th row with a match ${j}_{i}^{{\prime} }$ in *Z*-modality, then we call $(i,{j}_{i}^{{\prime} })$ a matched pair obtained via propagation. We can optionally filter out any matched pair via propagation in which the nearest-neighbor distance between ${[{\widetilde{Y}}_{{\mathtt{m}}}]}_{i\cdot }$ and ${[{\widetilde{Y}}_{{\mathtt{m}}}]}_{{j}_{i}\cdot }$ is above a user-specified threshold. The retained matched pairs compose the *Y*-to-*Z* propagated matching. This procedure is then repeated with the roles of *Y*- and *Z*-modalities switched to obtain the *Z*-to-*Y* propagated matching. Pooling all matched pairs from refined pivots and propagated matching together, we obtain a matching between meta-cells in *Y*-modality and those in *Z*-modality. Such a meta-cell-level matching defines a single-cell-level matching between the original datasets *Y* and *Z* by declaring $(i,{i}^{{\prime} })$ a matched pair for $1\le i\le {N}_{y},1\le {i}^{{\prime} }\le {N}_{z}$ if the meta-cell that *i* belongs to is matched to the meta-cell that ${i}^{{\prime} }$ belongs to.

*Scoring and directional pruning of matching*. For each single-cell-level matched pair $(i,{i}^{\prime} )$, we compute Pearson correlation between the *i*-th row of ${Y{}^{\mathtt{e}}}$ and the ${i}^{{\prime} }$-th row of ${Z^{\mathtt{e}}}$ (that is, corresponding rows in final joint embedding) as its matching score. We use these matching scores to prune single-cell-level matching, with the direction of pruning specified by the user. Suppose the user wants to find for each cell in *Z* a match in *Y* (for example, *Z* is a CODEX dataset and *Y* snRNA-seq). Then for each cell index $1\le {i}^{{\prime} }\le {N}_{z}$, we first list all refined pivots and propagated matching pairs that contain ${i}^{{\prime} }$. If the list is nonempty, we only retain the pair with the highest matching score. Otherwise, we declare no match for cell ${i}^{{\prime} }$ in *Z*-modality. If the direction is reversed, we apply the foregoing procedure with the roles of *Y* and *Z* switched. Furthermore, if no directional pruning is desired, we just keep all refined pivots and postscreening propagated matching pairs in the final single-cell matching. In Extended Data Figs. 3 and 4 and Supplementary Figs. 1 and 2, we benchmarked how evaluation metrics change with different choices of filtering proportions in propagation and in pruning. In Supplementary Table 4, we reported the filtering proportions used in the data analyses reported in this work. After filtering, propagation and potential pruning, the final list of matched pairs corresponds to the final matching in Fig. 1c.

### Systematic benchmarking on ground-truth datasets

#### MaxFuse and other methods in comparison

MaxFuse was implemented in Python, and the four methods used for comparison, Seurat V3, Harmony, Liger and BindSC, were implemented in R. All benchmarking datasets were preprocessed in the same way for all methods, including filtering of low-quality cells, selection of highly variable genes and protein features to be used in integration, feature linkage scheme (for example, protein to their corresponding gene names) and normalization of raw observed values (except for Liger which required scaling without centering). We used the default tuning parameters in each method suggested by the respective tutorial, with the exception of BindSC, for which we used the separate set of parameters suggested for the integration of protein-related data by its method tutorial website. For MaxFuse, initial matching used features that are weakly linked (for example, protein CD4 and RNA *CD4*) and are smoothed by all-feature nearest-neighbor graphs. For refined matching, all features from both modalities were used (for example, all proteins and RNAs that are highly variable). For other methods in comparison, BindSC used both the weakly linked features and all features, whereas others only used the weakly linked features by design. The full details were recorded and can be reproduced, with code deposited to https://github.com/shuxiaoc/maxfuse/tree/main/Archive.

#### Evaluation metrics

1. Cell-type matching accuracy: To evaluate the matching performance for Seurat V3, Liger, Harmony and BindSC, we used the respective integration embedding vectors produced by each method. For these methods, for each cell in one modality, we regarded its nearest neighbor from the other modality under Pearson correlation distance in the embedding space as its match. For MaxFuse, we directly used matched pairs produced in the final result. For all methods, we use the same matching direction (for example, for each cell in CODEX data finding a matched cell in scRNA-seq data) for fair comparison. Accuracy of the matchings was measured by fraction of matched pairs with identical cell-type annotations. Details on cell-type annotation are given below in the description of each benchmarking dataset.
2. FOSCTTM: FOSCTTM was used to evaluate single-cell-level alignment accuracy on datasets with ground-truth single-cell-level pairing. The measure has been used previously in cross-modality alignment benchmarking tasks^19,36,37^. For such data, *N*_*y*_ = *N*_*z*_ = *N*, and FOSCTTM is defined as:
  $${{{\rm{FOSCTTM}}}}=\frac{1}{2N}\left(\mathop{\sum }\limits_{i=1}^{N}\frac{{n}_{y}^{(i)}}{N}+\mathop{\sum }\limits_{i=1}^{N}\frac{{n}_{z}^{(i)}}{N}\right),$$
  where for each $i,{n}_{y}^{(i)}=\left\vert \{\,j\left.\right\vert d({\,y}_{i},{z}_{j}) < d({\,y}_{i},{z}_{i})\}\right\vert$ with *d* a distance metric in the joint embedding space and for *l* = 1, …, *N*, *y*_*l*_ and *z*_*l*_ are the embedded vectors of the *l*-th cell with its measurements in *Y*- and *Z*-modality, respectively. The counts ${n}_{z}^{(i)},i=1,\ldots ,N$, are defined analogously. A lower value of FOSCTTM indicates better integration performance.
3. FOSKNN: FOSKNN was used to evaluate single-cell-level alignment accuracy on datasets with ground-truth single-cell-level pairing. For such data, *N*_*y*_ = *N*_*z*_ = *N*. For any method in comparison, let {*y*_*i*_: *i* = 1, …, *N*} be the coordinates of cells in the joint embedding space from their *Y*-modality information, and let {*z*_*i*_: *i* = 1, …, *N*} be embedding coordinates from their *Z*-modality information. Then
  $${{{\rm{FOSKNN}}}}=\frac{1}{2N}\left(\mathop{\sum }\limits_{i=1}^{N}{{{{\bf{1}}}}}_{{E}_{y,k}}^{(i)}+\mathop{\sum }\limits_{i=1}^{N}{{{{\bf{1}}}}}_{{E}_{z,k}}^{(i)}\right)$$
  where for $i=1,\ldots ,N,{{{{\bf{1}}}}}_{{E}_{y,k}}^{(i)}$ is the indicator of whether the *k* closest embedded vectors from *Z*-modality to *y*_*i*_ includes *z*_*i*_. The quantity ${{{{\bf{1}}}}}_{{E}_{z,k}}^{(i)}$ is defined analogously. A higher value of FOSKNN indicates better integration performance.
4. Silhouette F1 score: Silhouette F1 score has been used to simultaneously measure modality mixing and information preservation post integration process^21,35^. In brief, the F1 score was calculated by 2 ⋅ slt_mix ⋅ slt_clust/(slt_mix + slt_clust), where slt_mix is defined as one minus normalized Silhouette width with the label being modality index (two modalities); slt_clust is defined by the normalized Silhouette width with the label being cell-type annotations (for example, ‘CD4 T’, ‘CD8 T’, ‘B’ and so on). All Silhouette widths were computed using the silhouette function from R package cluster.
5. ARI F1 score: ARI F1 score has been used to jointly measure modality mixing and information preservation post integration process^21,35^. The score was calculated in a similar way to Silhouette F1 score, while the ARI was used instead of the Silhouette width. All ARI scores were computed using the function adjustedRandIndex in R package mclust.

#### CITE-seq PBMC dataset analysis

The CITE-seq data from human PBMCs with antibody panel of 228 markers were retrieved from Hao et al.^33^ and cell-type annotations (level 1: 8 cell types; and level 2: 31 cell types) were directly retrieved from the original annotation in ref. ^33^. For benchmarking purposes, five batches of cells, each with 10,000 cells, were randomly sampled from the original dataset and used for benchmarking. The first 15 components of the embedding vectors produced by all methods were used for benchmarking metric calculation. The UMAP visualization of the integration process was also calculated with the first 15 components of the embedding vectors. For visualization purposes, the 31 cell types of level 2 annotation were manually binned into 20 cell types in the UMAP cell-type coloring.

For analyses with fewer antibodies, we ranked the importance of each individual antibody in the panel in terms of phenotyping contribution. The importance score was calculated by training a random forest model (function randomForest in R package randomForest, with default parameters) using all antibodies to predict cell-type labels (annotation level 2), then a permutation feature importance test (function varImp with default parameters in R package caret) was performed on the trained model to acquire the importance scores. Then antibodies were ranked by the importance scores, and four panels were used for the antibody dropping test: (1) full 228-antibody panel; (2) top 100 most important antibodies; (3) top 50 most important antibodies; (4) top 30 most important antibodies.

#### CITE-seq bone marrow cell dataset analysis

The CITE-seq healthy human bone marrow cells (BMCs) data with an antibody panel of 25 markers were retrieved from the R package SeuratData ‘bmcite’; these data were also reported by Hao et al.^33^. A total of 20,000 cells were randomly sampled from the original dataset and used for benchmarking. The first 15 components of the embedding vectors produced by all methods were used for benchmarking metric calculation. The UMAP visualization of the integration process was also calculated with the first 15 components of the embedding vectors. The original cell-type annotation (lv2) from the R package was binned into eight populations, ‘DC’, ‘progenitor’, ‘monocyte’, ‘NK’, ‘B’, ‘CD4 T’, ‘CD8 T’ and ‘Other T’, and used for benchmarking.

#### Abseq BMC dataset analysis

The Abseq healthy human BMC data with antibody panel of 97 markers and whole transcriptome sequencing were retrieved from Triana et al.^39^. All cells in the dataset (~13,000), except cells belonging to cell types with insufficient numbers of cells (<50 cells, annotated as ‘Doublet and Triplets’, ‘Early GMP’, ‘Gamma delta T cells’, ‘Immature B cells’, ‘Metaphase MPPs’, ‘Neutrophils’ in ref. ^39^), were included for integration. The remaining 14 cell types were used during benchmarking. The first 15 components of the embedding vectors produced by all methods were used for benchmarking metric calculation. The UMAP visualization of the integration process was also calculated with the first 15 components of the embedding vectors.

#### TEA-seq PBMC dataset analysis

The TEA-seq neutrophil-depleted human PBMC dataset was retrieved from Swanson et al.^41^ (GSM4949911). This dataset contains 46 antibodies and chromatin accessibility information. Cell-type annotation was performed using R package Seurat (v.4) WNN-multi-modal clustering pipeline: function FindMultiModalNeighbors was run on the antibody-derived tags (ADT) assay principal component analysis (PCA) output (first 25 components) and the ATAC assay latent semantic indexing (LSI) output (first 2–50 components, calculated by R package Archr^42^). Subsequently, the function FindClusters was used to generate unsupervised clustering (with parameters algorithm = 3, resolution = 0.2), followed by manual annotation. A total of eight populations were identified (‘Naive CD4’, ‘Mem CD4’, ‘Monocyte’, ‘NK’, ‘Naive CD8’, ‘Mem CD8’, ‘Effector CD8’, ‘B’, ‘NK’), and the total number of cells was ~7,400. ADT expressions and gene activity scores (calculated by R package Archr^42^) were used as input for MaxFuse and other methods. Additionally, during matching refinement, MaxFuse used LSI reductions of the ATAC peaks (first 2–50 components) as features for the ATAC modality. The first 15 components of the embedding vectors produced by all methods were used for benchmarking metric calculation. The UMAP visualization of the integration process was also calculated with the first 15 components of the embedding vectors.

#### ASAP-seq PBMC dataset analysis

The ASAP-seq healthy human PBMC data (CD28 and CD3 stim PBMC control group) with an antibody panel of 227 markers and chromatin accessibility information were retrieved from Mimitou et al.^40^ (GSM4732109 and GSM4732110). Cell-type annotation was performed using R package Seurat (v.4) WNN-multi-modal clustering pipeline: the function FindMultiModalNeighbors was run on ADT PCA (first 18 components) and ATAC LSI (2–40 components, calculated by R package Archr). Subsequently, the function FindClusters was used to generate unsupervised clustering (with parameters algorithm = 3, resolution = 0.3), followed by manual annotation. A total of nine populations were identified (‘Naive CD4’, ‘Mem CD4’, ‘Monocyte’, ‘NK’, ‘Naive CD8’, ‘Mem CD8’, ‘B’, ‘Other T’, ‘dirt’), and ‘dirt’ was removed from subsequent analyses, resulting in about 4,400 cells used. ADT expressions and gene activity scores (calculated by R package Archr) were used as input for MaxFuse and other methods. Additionally, during matching refinement, MaxFuse used LSI reductions of the ATAC peaks (first 2–50 components) as features for the ATAC modality. The first 15 components of the embedding vectors produced by all methods were used for benchmarking metric calculation. The UMAP visualization of the integration process was also calculated with the first 15 components of the embedding vectors.

### MaxFuse on spatial-omics matching

#### CODEX and scRNA-seq human tonsil dataset analysis

CODEX multiplex imaging data of human tonsil tissues with a panel of 46 antibodies were retrieved from Kennedy-Darling et al.^49^. Images from tonsil-9338 (region X2-8, Y7-15) were used. Whole-cell segmentation was performed with a local implementation of Mesmer^66^, with weights downloaded from: https://deepcell-data.s3-us-west-1.amazonaws.com/model-weights/Multiplex_Segmentation_20200908_2_head.h5. Inputs of segmentation were DAPI (nuclear) and CD45 (membrane). Signals from the images were capped at 99.7th percentile, with prediction parameter model_mpp = 0.8. Cells smaller than 30 pixels or larger than 800 pixels were excluded. Signals from individual cells were then extracted, and scaled to the [0, 1] interval, with percentile cutoffs at 0.5% (floor) and 99.5% (ceiling). Cell-type annotation was performed using R package Seurat clustering pipeline: the function FindNeighbors was run on CODEX protein PCA (first 15 components). Subsequently, the function FindClusters was used to generate unsupervised clustering (with parameter resolution = 1), followed by manual annotation. A total of ten populations were identified (‘B-CD22-CD40’, ‘B-Ki67’, ‘Plasma’, ‘CD4 T’, ‘CD8 T’, ‘DC’, ‘Fibro/Epi’, ‘Vessel’, ‘Other’ and ‘Dirt’), and six populations (~180,000 cells in total) were used in subsequent analyses (‘B-CD22-CD40’, ‘B-Ki67’, ‘Plasma’, ‘CD4 T’, ‘CD8 T’ and ‘DC’).

scRNA-seq data of dissociated human tonsil cells were retrieved from King et al.^50^. The preprocessing and cell typing steps were done in the R package Seurat, following the description presented in ref. ^50^. In brief, tonsil cells (‘t1’, ‘t2’ and ‘t3’) were merged, then filtered by the criteria nFeature_RNA > 200 & nFeature_RNA < 7500 & percent.mt < 20, and subsequently values were normalized by the function SCTransform. Harmony batch correction was performed for different tonsils for clustering only, with the function RunHarmony. Unsupervised clustering was performed by the function FindNeighbors with Harmony embedding (1–27 dimensions) and function FindClusters with resolution = 0.5. A total of eight populations were defined (‘B-CD22-CD40’, ‘B-Ki67’, ‘circulating B’, ‘Plasma’, ‘CD4 T’, ‘CD8 T’, ‘DC’, ‘Other’), and six populations (~13,000 cells in total) were used in subsequent analyses (‘B-CD22-CD40’, ‘B-Ki67’, ‘Plasma’, ‘CD4 T’, ‘CD8 T’ and ‘DC’).

Boundaries of GCs from the CODEX images were drawn manually, and dilation and erosion from the boundary was performed with the Python package skimage, with functions morphology.binary_dilation and morphology.disk. Ten layers inward and ten layers outward from the boundary (each layer = 30 pixels; resolution: 376 nm per pixel) were performed, respectively. Cells were assigned to each layer based on locations of centroids. The RNA expression levels from each layer, based on the averaged CODEX-matched scRNA-seq cells, were plotted with the R package ggplot2. The UMAP visualization of the integration process was calculated with the first 15 components of the embedding vectors.

#### HUBMAP atlas: tri-modal human intestine dataset analysis

CODEX multiplex imaging (48 markers), snRNA-seq and snATAC-seq data of healthy human intestine cells were acquired from Hickey et al.^31^. For CODEX, samples ‘B005_SB’ and ‘B006_CL’ were used, while for snRNA-seq and snATAC-seq, single-ome sequencing data of four donors (‘B001’, ‘B004’, ‘B005’, ‘B006’) from the study were used. Cells annotated as ‘B cells’, ‘T cells’, ‘Endothelial’, ‘Enteroendocrine’, ‘Goblet’, ‘Mono_Macrophages’, ‘Plasma’, ‘Smooth muscle’ and ‘Stroma’ were selected for the integration process. Cell counts for each modality used for MaxFuse were: CODEX ~100,000 (small bowel) and ~70,000 (colon); snRNA-seq ~32,000 (small bowel) and ~16,000 (colon); snATAC-seq ~28,000 (small bowel) and ~21,000 (colon). CODEX protein expressions, snRNA-seq RNA expressions, snATAC-seq gene activity scores and LSI scores (calculated with R package Archr) were used as MaxFuse input (RNA expressions, gene activity scores and LSI scores were batch-corrected by Harmony^20^, based on patient ID). The matching and integration processes were done on colon and small bowel samples, respectively.

Pairwise MaxFuse alignments of cells between protein (CODEX) and RNA (snRNA-seq), and of cells between RNA (snRNA-seq) and ATAC (snATAC-seq), were performed. Refined pivots from the two bimodal alignments were chained together by using the pivot cells in the RNA modality as the intermediary, resulting in a list of tri-modal pivots linking all three modalities. Subsequently, we used these pivots to calculate a tri-omic embedding via gCCA^21,59^. In particular, we used the gCCA formulation and algorithm described in ref. ^21^.

The UMAP visualization of the tri-modal integration was calculated with the first 15 components of the embedding vectors (gCCA scores in this case). Embeddings of CODEX cells were overlaid with their protein expressions, or their matched cells’ RNA expressions, or gene activity scores. Spatial locations of these expression values and scores were plotted based on CODEX cells’ *x*–*y* centroid locations. Additionally, we showed spatial locations of transcription factor motif enrichment scores (*Z*-score) of CODEX cells, based on their matched snRNA-seq cells, which were calculated by the R package chromVAR^60^. All values were capped between 5% and 95% quantiles for visualization purposes during plotting.

### Reporting summary

Further information on research design is available in the Nature Portfolio Reporting Summary linked to this article.

## Online content

Any methods, additional references, Nature Portfolio reporting summaries, source data, extended data, supplementary information, acknowledgements, peer review information; details of author contributions and competing interests; and statements of data and code availability are available at 10.1038/s41587-023-01935-0.

## Supplementary information

Supplementary Information Supplementary Figs. 1–13, material and methods, and Tables 1–5.

Reporting Summary

## Data Availability

All data used in this manuscript are publicly available. The links are listed here: CITE-seq PBMC from Hao et al.^33^: https://atlas.fredhutch.org/data/nygc/multimodal/pbmc_multimodal.h5seurat; CITE-seq BMC from Hao et al.^33^: https://satijalab.org/seurat/articles/multimodal_reference_mapping.html (file: ‘bmcite’ with ’SeuratData’); Ab-seq BMC from Triana et al.^39^: https://figshare.com/articles/dataset/Expression_of_97_surface_markers_and_RNA_transcriptome_wide_in_13165_cells_from_a_healthy_young_bone_marrow_donor/13397987; TEA-seq PBMC from Swanson et al.: ncbi.nlm.nih.gov/geo/query/acc.cgi?acc=GSM4949911; ASAP-seq PBMC from Mimitou et al.^40^: https://www.ncbi.nlm.nih.gov/geo/query/acc.cgi?acc=GSE156473 (GSM4732109 and GSM4732110); CODEX tonsil from Kennedy et al.^49^: https://onlinelibrary.wiley.com/doi/10.1002/eji.202048891; scRNA-seq tonsil from King et al.^50^: https://www.ncbi.nlm.nih.gov/geo/query/acc.cgi?acc=GSE165860 (tonsil 1a, 1b, 2a, 2b, 3a, 3b); Multiome (scRNA-seq and scATAC-seq) retina from Wang et al.^48^: https://www.ncbi.nlm.nih.gov/geo/query/acc.cgi?acc=GSM5866073; Multiome (scRNA-seq and scATAC-seq) PBMC from 10x Genomics datasets^46^: https://www.10xgenomics.com/resources/datasets (PBMC from a Healthy Donor - Granulocytes Removed Through Cell Sorting (10k)); Multiome (scRNA-seq and scATAC-seq) mouse E18 from 10x Genomics datasets^46^: https://www.10xgenomics.com/resources/datasets (Fresh Embryonic E18 Mouse Brain (5k)); Multiome (scRNA-seq and scATAC-seq) cerebral cortex from Trevino et al.^47^: https://www.ncbi.nlm.nih.gov/geo/query/acc.cgi?acc=GSE162170 (multiome samples).

## Code availability

All code used in this study, including the MaxFuse software and the analysis code, can be found at https://github.com/shuxiaoc/maxfuse.
