# Supplementary material for: Integration of spatial and single-cell data across modalities with weakly linked features
Source: Nat Biotechnol. 2023 Sep 7;42(7):1096–106. doi: 10.1038/s41587-023-01935-0 (PMC11638971; doi:10.1038/s41587-023-01935-0)
Supplement: Supplementary file 1 — Supplementary Figs. 1–13, material and methods, and Tables 1–5. [file 41587_2023_1935_MOESM1_ESM.pdf]

nature biotechnology

Article https://doi.org/10.1038/s41587-023-01935-0

Integration of spatial and single-cell data across modalities with weakly linked features

In the format provided by the authors and unedited

nature biotechnology

Article https://doi.org/10.1038/s41587-023-01935-0

Integration of spatial and single-cell data across modalities with weakly linked features

In the format provided by the authors and unedited

Supplementary notes for “Integration of spatial and single-cell data across modalities with weakly linked features”

Shuxiao Chen[1,*], Bokai Zhu[2,3,*], Sijia Huang[1], John W. Hickey[3], Kevin Z. Lin[4], Michael Snyder[5], William J. Greenleaf[5], Garry P. Nolan[2,3,†], Nancy R. Zhang[1,†], and Zongming Ma[6,†]

[1]Department of Statistics and Data Science, The Wharton School, University of Pennsylvania, PA, United States
[2]Department of Microbiology and Immunology, Stanford University, Stanford, CA, United States
[3]Department of Pathology, Stanford University, Stanford, CA, United States
[4]Department of Biostatistics, University of Washington, Seattle, WA, United States
[5]Department of Genetics, Stanford University, Stanford, CA, United States
[6]Department of Statistics and Data Science, Yale University, New Haven, CT, United States
[*]These authors contributed equally.
[†]These authors jointly supervised this work.

Correspondence: ***gnolan@stanford.edu, nzh@wharton.upenn.edu, zongming.ma@yale.edu.***

Supplementary Figures

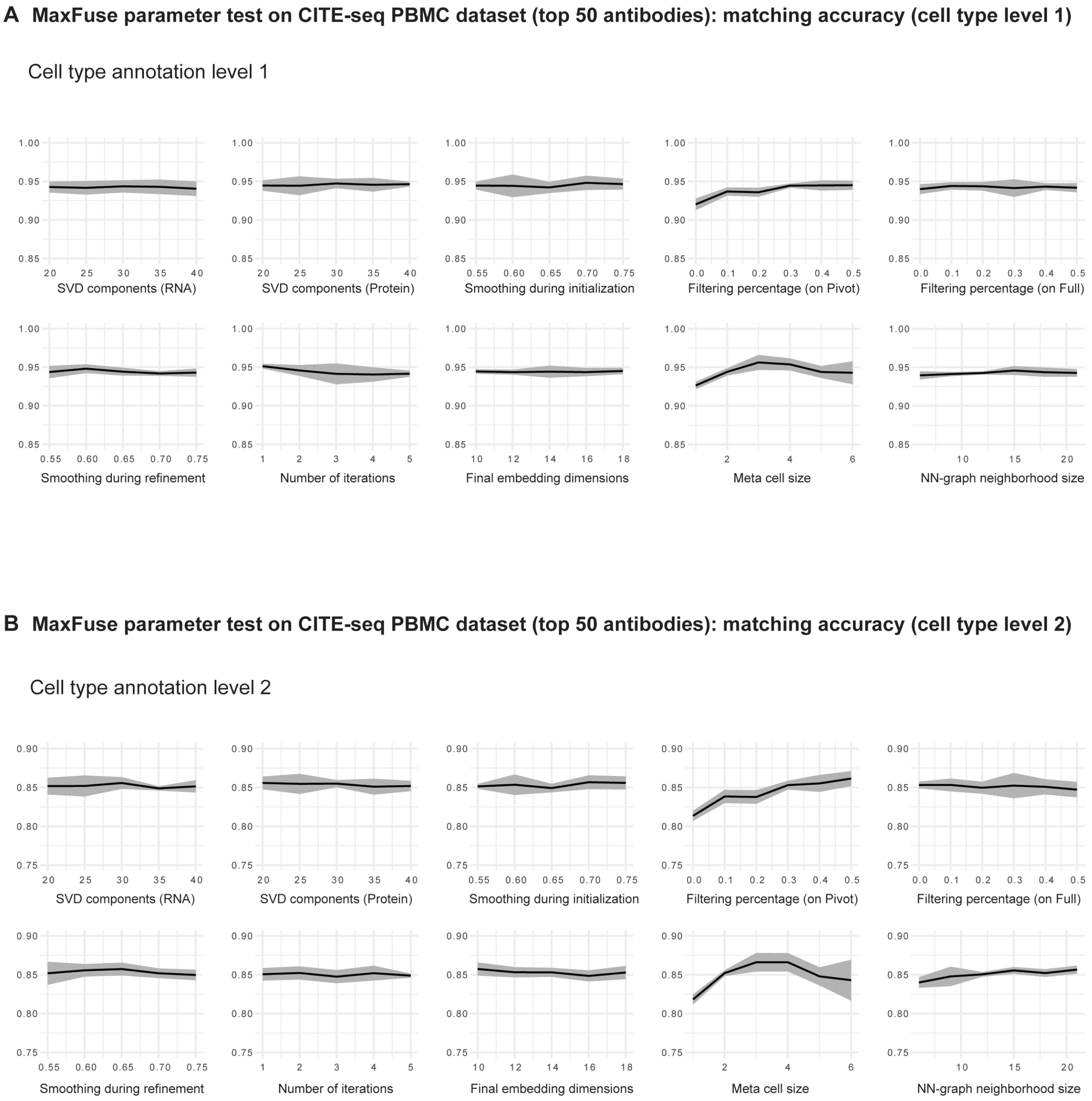

Figure S1: Benchmarking of robustness to tuning parameters in MaxFuse integration on CITE-seq PBMC with top 50 antibodies from Hao et al. (1), evaluated by matching accuracy at two levels. (A) Matching accuracy (cell type level 1) versus a range of SVD components for different modalities, smoothing weights during initialization and refinement, filtering percentages on pivot and on full matching, number of iterations, final CCA embedding dimensions, meta-cell size, and NN-graph neighborhood size. Line indicates mean value and shadow indicates 95% CI on both sides. **(B)** Matching accuracy (cell type level 2) versus a range of SVD components for different modalities, smoothing weights during initialization and refinement, filtering percentages on pivot and on full matching, number of iterations, final CCA embedding dimensions, meta-cell size, and NN-graph neighborhood size. Line indicates mean value and shadow indicates 95% CI on both sides.

A **MaxFuse parameter test on CITE-seq PBMC dataset (top 50 antibodies): FOSCTTM (smaller is better)**

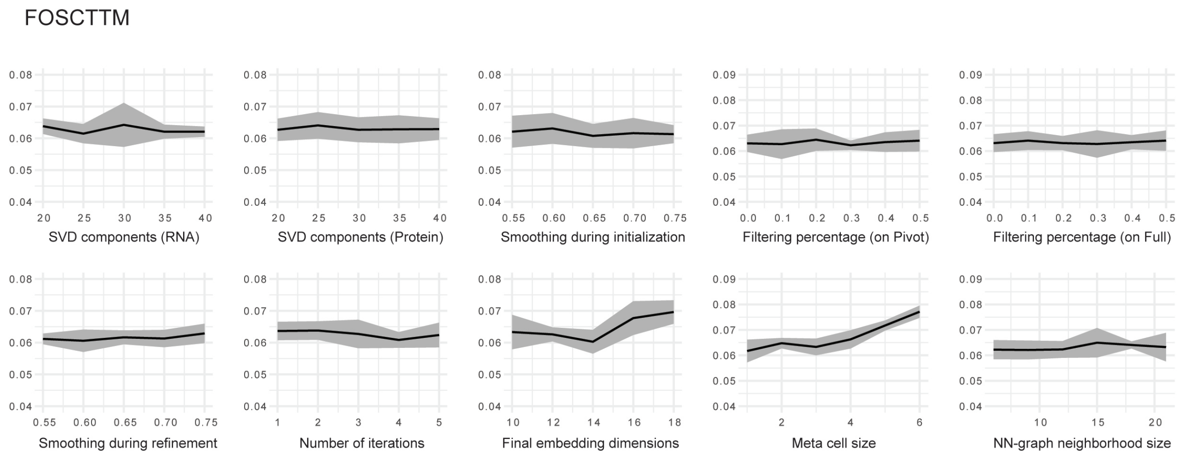

B **MaxFuse parameter test on CITE-seq PBMC dataset (top 50 antibodies): FOSKNN**

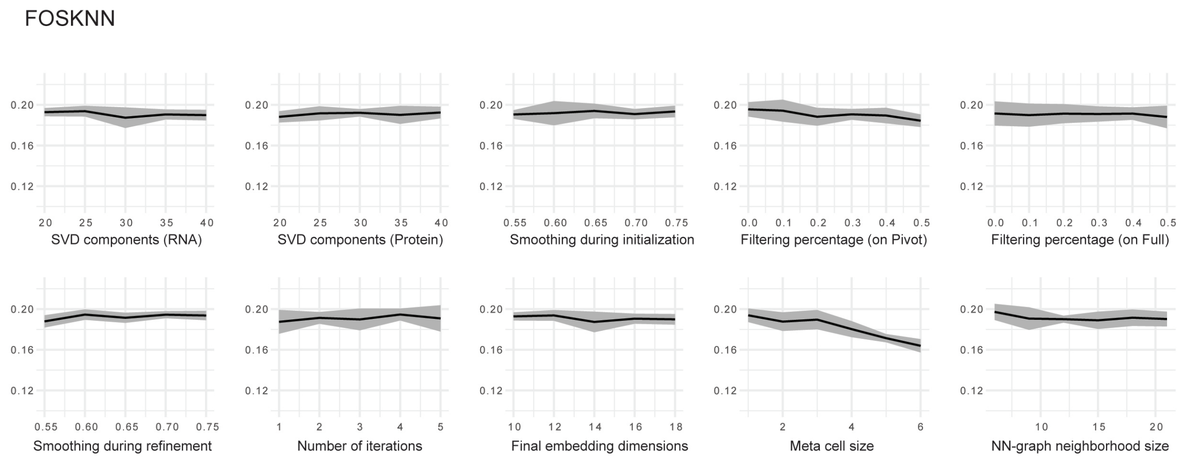

Figure S2: Benchmarking of robustness to tuning parameters in MaxFuse integration on CITE-seq PBMC with top 50 antibodies from Hao et al. (1), evaluated by FOSCTTM and FOSKNN. (A) FOSCTTM scores versus a range of SVD components for different modalities, smoothing weights during initialization and refinement, filtering percentages on pivot and on full matching, number of iterations, final CCA embedding dimensions, meta-cell size, and NN-graph neighborhood size. Line indicates mean value and shadow indicates 95% CI on both sides. **(B)** FOSKNN scores versus a range of SVD components for different modalities, smoothing weights during initialization and refinement, filtering percentages on pivot and on full matching, number of iterations, final CCA embedding dimensions, meta-cell size, and NN-graph neighborhood size. Line indicates mean value and shadow indicates 95% CI on both sides.

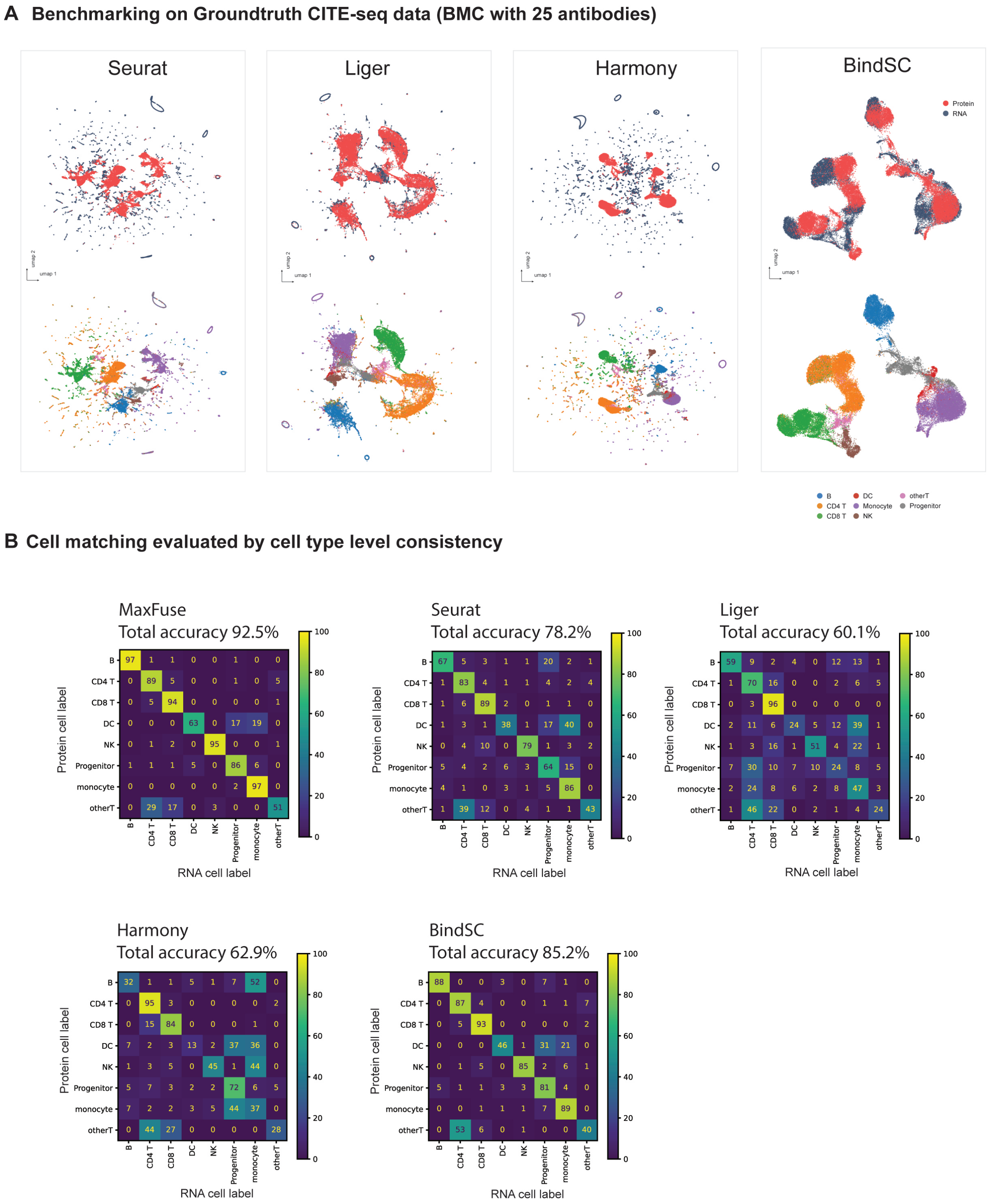

Figure S3: Benchmarking on ground-truth CITE-seq BMC data with 25 antibodies from Hao et al. (1). (A) UMAP visualization of Seurat (V3), Liger, Harmony, and BindSC integration results, colored by modality (upper panel) or cell types (lower panel). **(B)** Cell matching accuracy results (cell type level) of different methods.

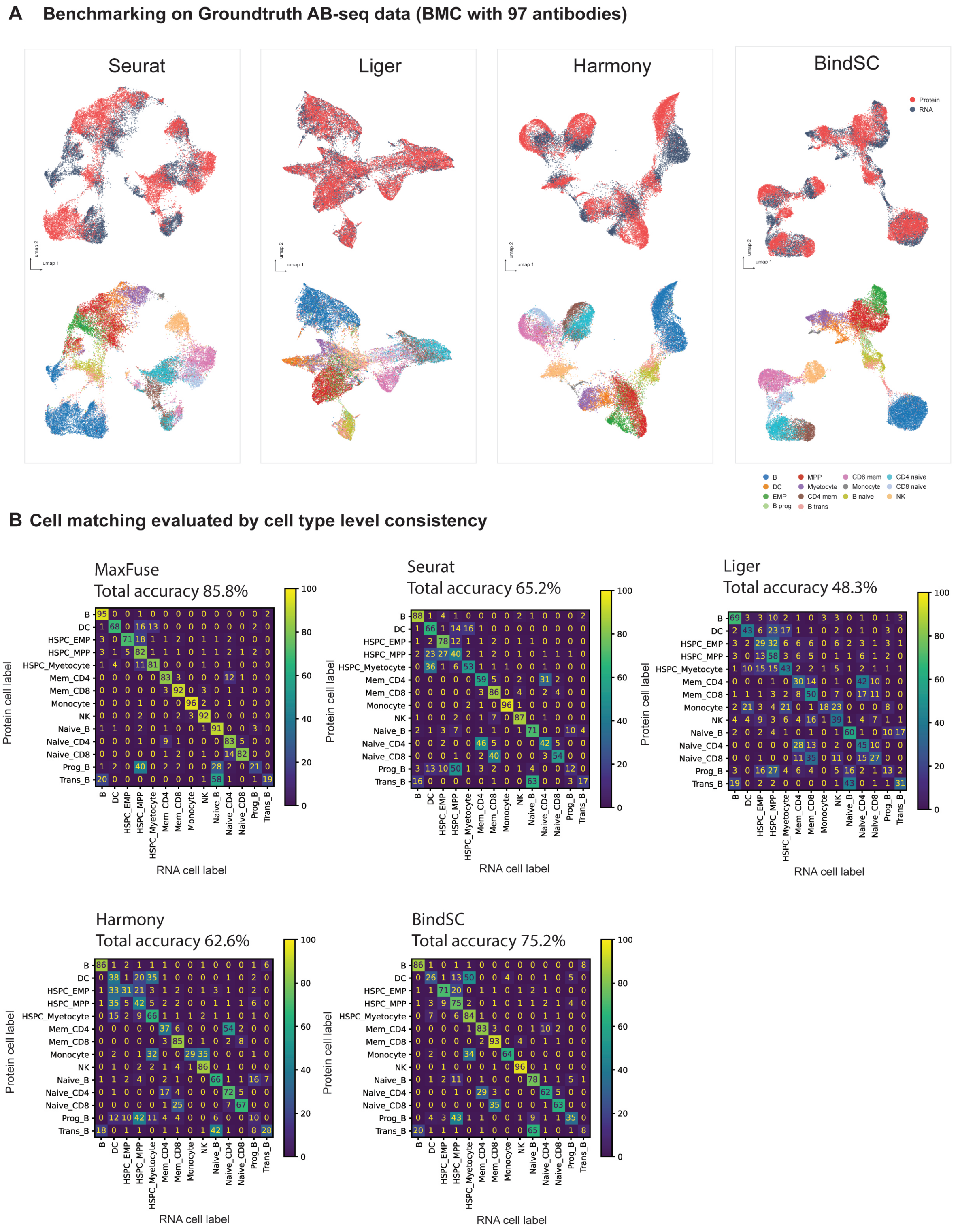

Figure S4: Benchmarking on ground-truth Abseq BMC data with 97 antibodies from Mimitou et al. (2). (A) UMAP visualization of Seurat (V3), Liger, Harmony, and BindSC integration results, colored by modality (upper panel) or cell types (lower panel). **(B)** Cell matching accuracy results (cell type level) of different methods.

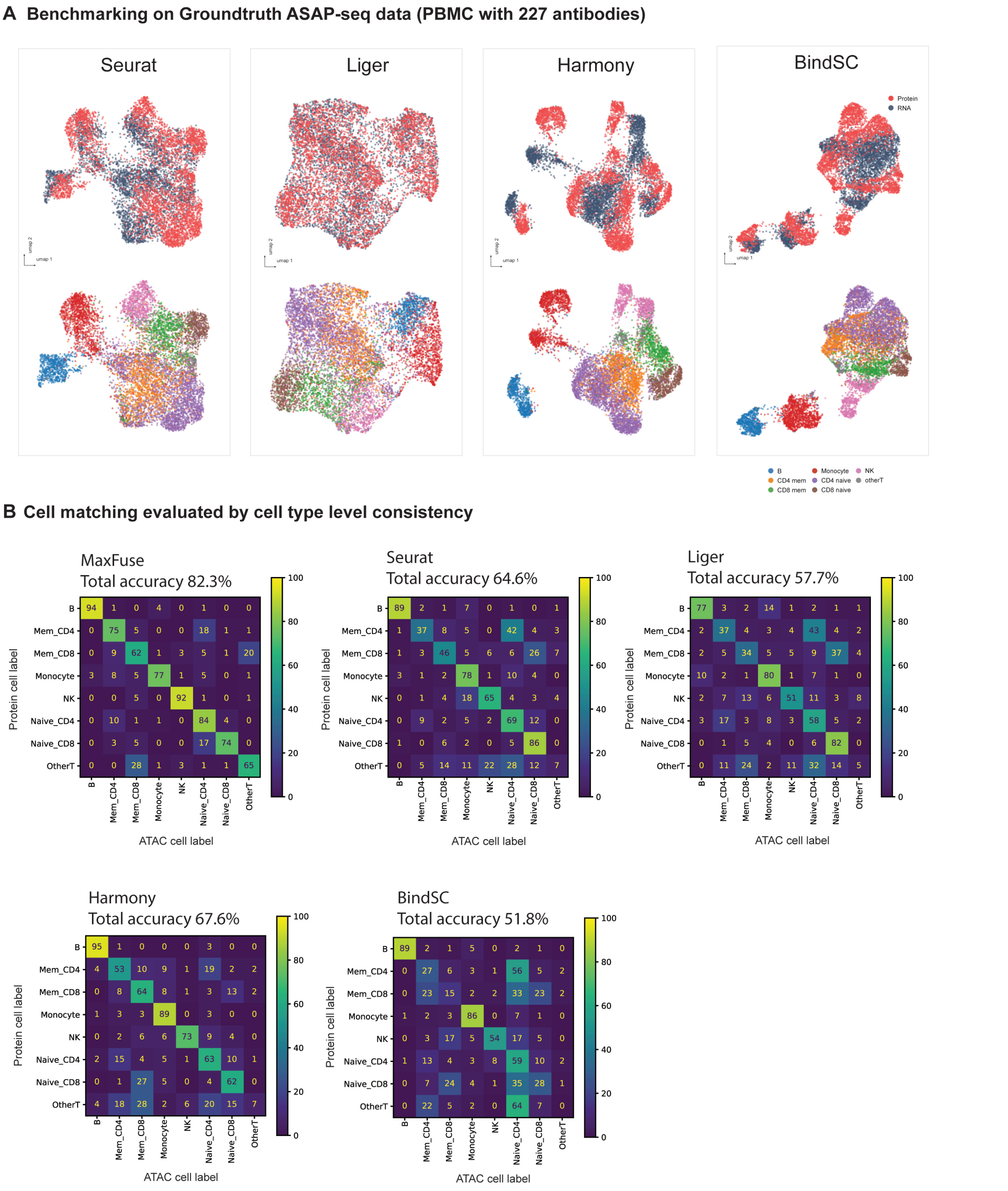

Figure S5: Benchmarking on ground-truth ASAP-seq PBMC data with 227 antibodies from Triana et al. (3). (A) UMAP visualization of Seurat (V3), Liger, Harmony, and BindSC integration results, colored by modality (upper panel) or cell types (lower panel). **(B)** Cell matching accuracy results (cell type level) of different methods.

A Benchmarking on Groundtruth TEA-seq data (PBMC with 46 antibodies)

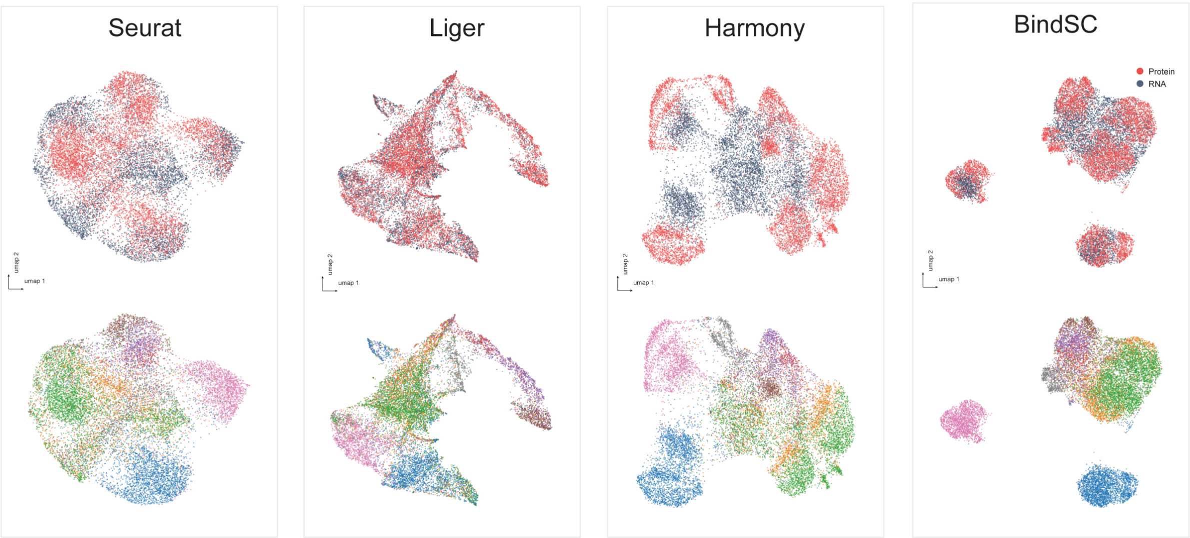

B Cell matching evaluated by cell type level consistency

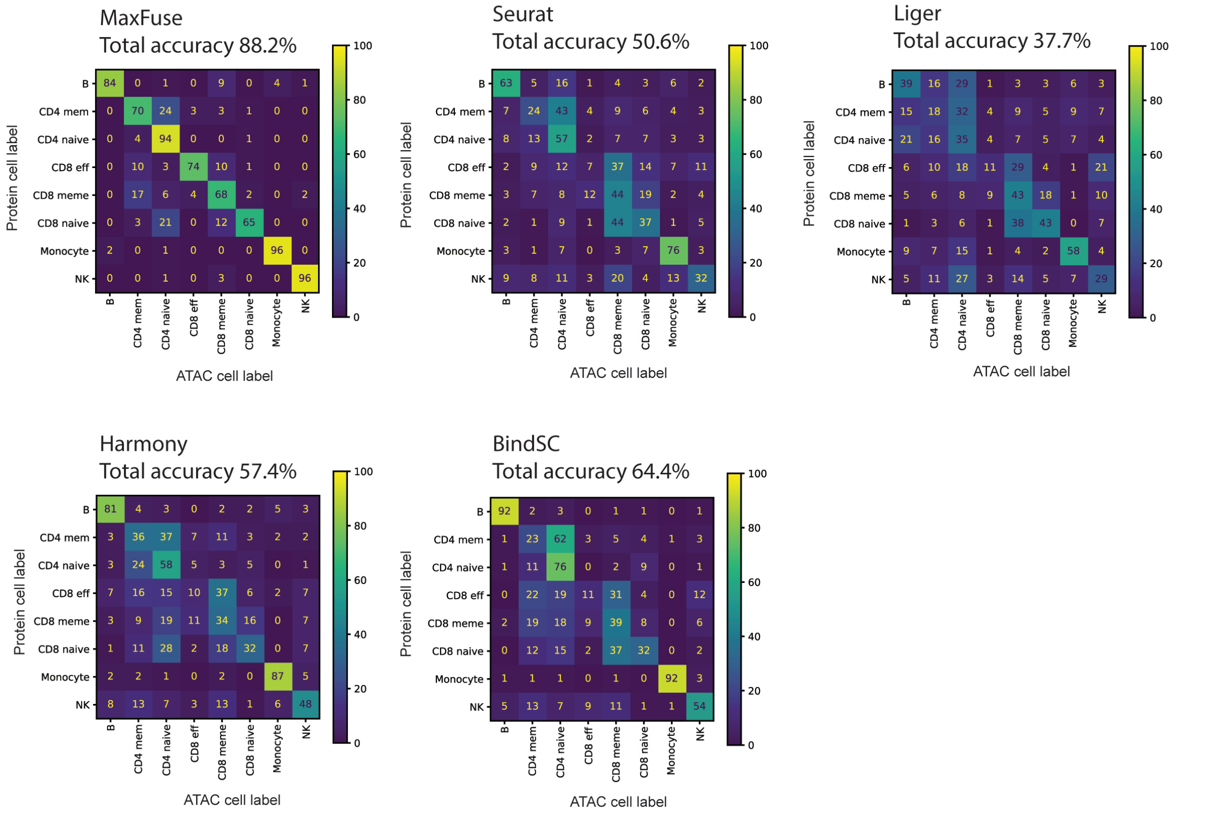

Figure S6: Benchmarking on ground-truth TEA-seq PBMC data with 46 antibodies from Swanson et al. (4). (A) UMAP visualization of Seurat (V3), Liger, Harmony, and BindSC integration results, colored by modality (upper panel) or cell types (lower panel). **(B)** Cell matching accuracy results (cell type level) of different methods.

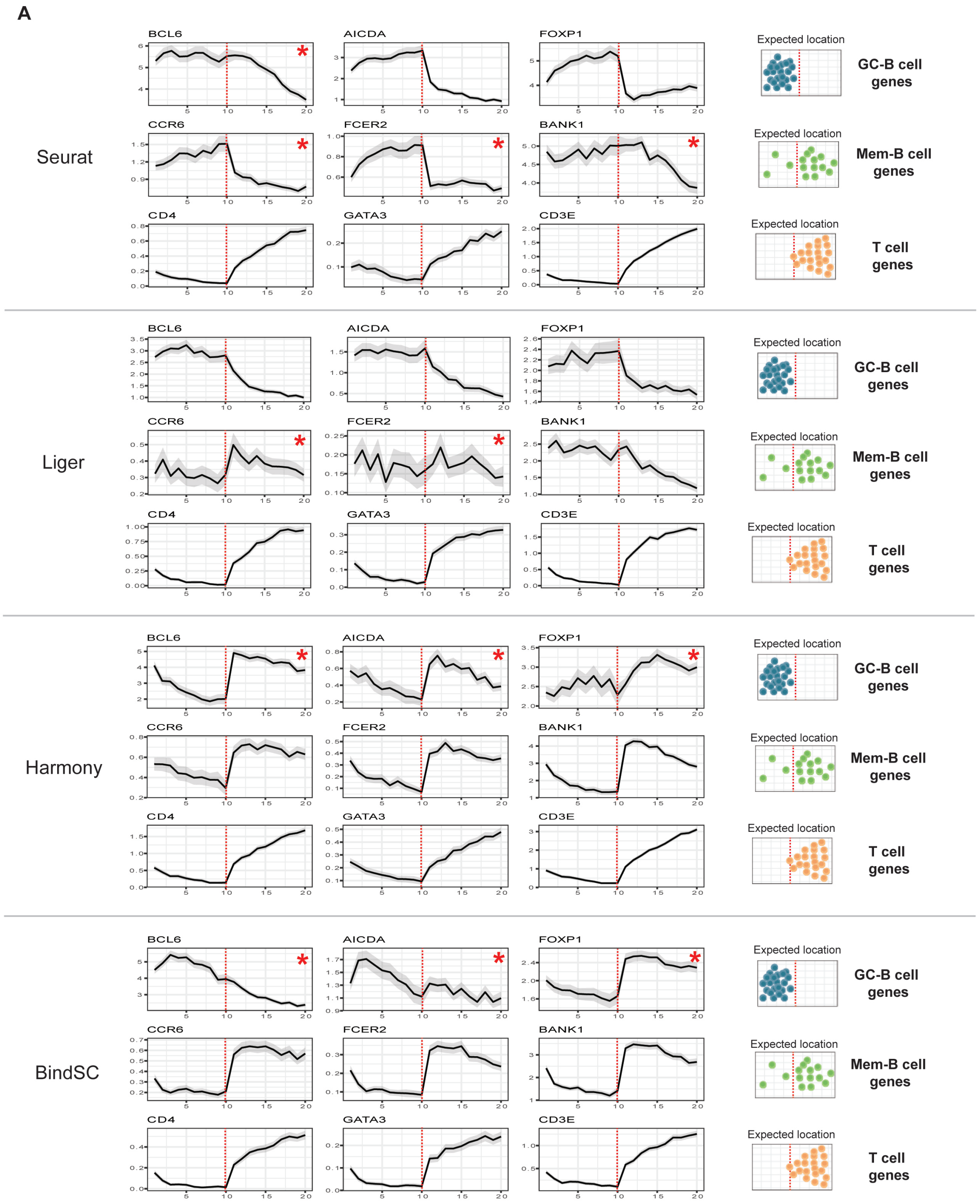

Figure S7: RNA expression patterns recovered by matching results provided by other methods on human tonsil CODEX and scRNA-seq data from Kennedy-Darling et al. (5) and King et al. (6). (A) Single-cell level matching information was retrieved from each method, creating a CODEX-scRNAseq multiome data. Subsequently, the average matched RNA expression level within each CODEX spatial layer (defined in Figure 4) was plotted. Each line indicates mean value with shadow area covering 95% CI for the mean at each position. Red stars indicate spurious prediction of spatial RNA distribution from integration that is not supported by known biology.

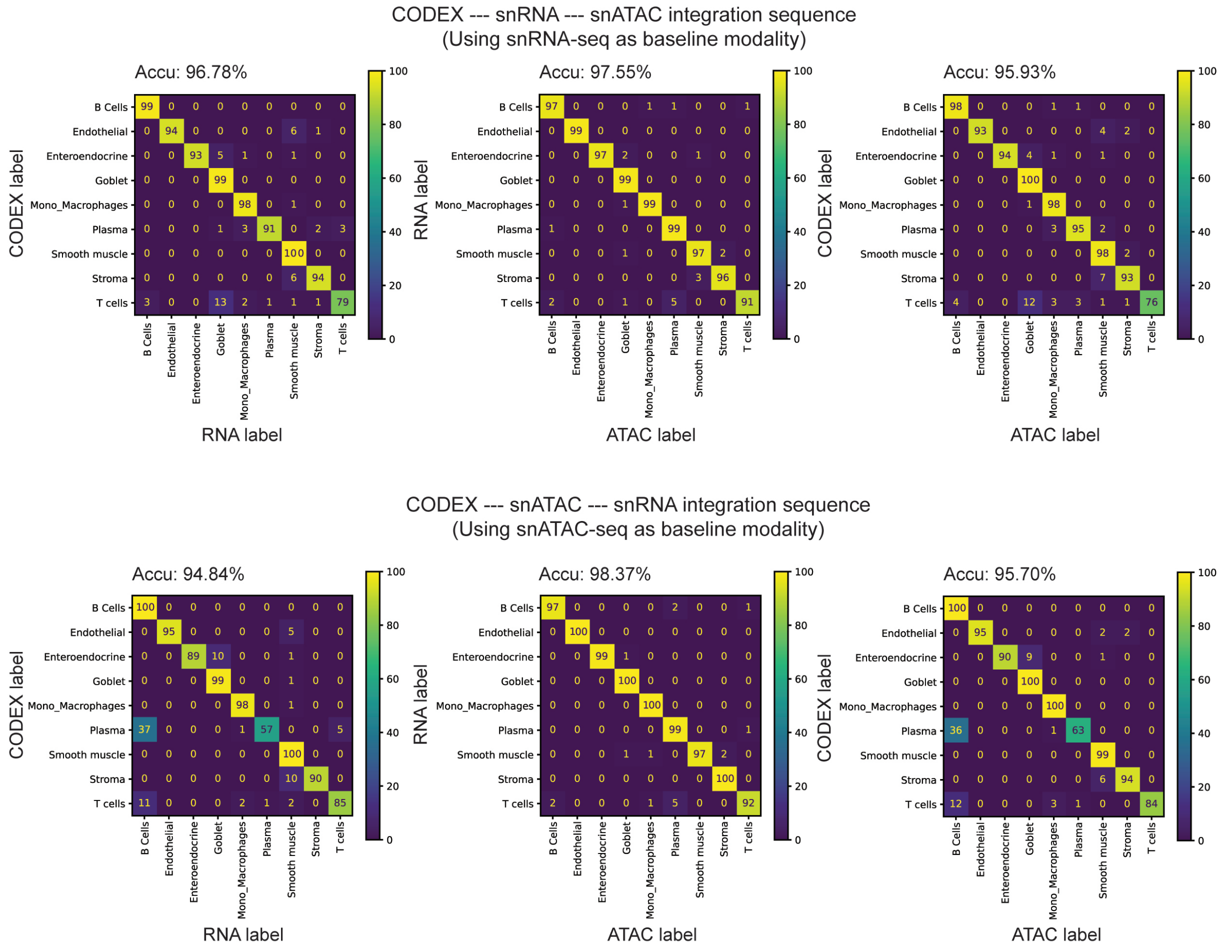

Figure S8: Comparison of tri-modal integration quality using different baseline modality. HUBMAP colon data (7) tri-modal (CODEX, snRNA-seq, snATAC-seq) integration by MaxFuse with different baseline modalities. One modality was chosen as baseline. The other two modalities were first integrated with the baseline individually, and then chained based on their integration with the baseline (See Methods for detail). Confusion matrices of cell type annotation matching accuracy were plotted for each pair of modalities. Upper panel: results with snRNA-seq dataset as baseline; Lower panel: results with snATAC-seq dataset as baseline.

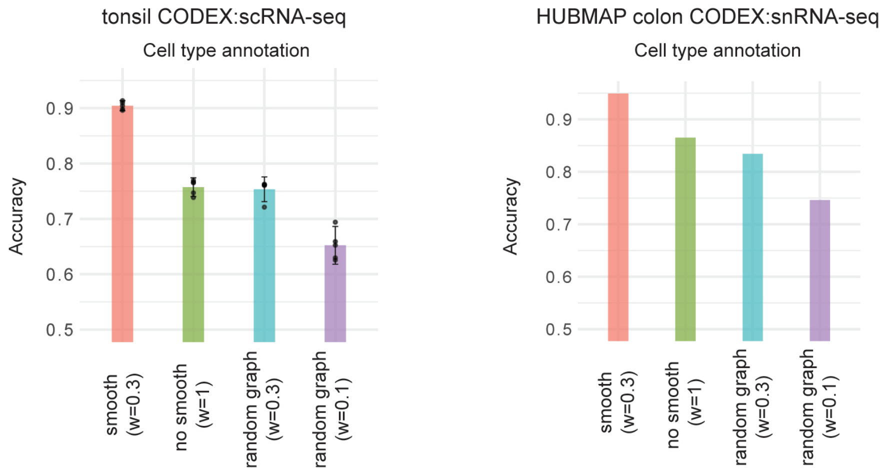

Figure S9: Benchmarking of fuzzy smoothing effects. (Left) Tonsil CODEX & scRNA-seq (5, 6) integration by MaxFuse using different smoothing settings on 5 random batches (each with 10,000 scRNA-seq cells and 30,000 CODEX cells): with smoothing (w = 0.3) during initialization and refinement (setting used in producing Figure 4); no smoothing (w = 1) during initialization and refinement; smoothing performed on a randomly constructed graph (obtained from randomly assigning neighbors) with smoothing weight w = 0.3; smoothing performed on a randomly constructed graph (obtained from randomly assigning neighbors) with smoothing weight w = 0.1. The barplot of cell type matching accuracy shows mean value with 95% CI for each setting, with raw values from five random samples plotted as dots. **(Right)** HUBMAP colon CODEX & snRNA-seq (7) integration by MaxFuse using different smoothing settings: with smoothing (w = 0.3) during initialization and refinement; no smoothing (w = 1) during initialization and refinement; smoothing performed on a randomly constructed graph (w = 0.3); smoothing performed on a randomly constructed graph (w = 0.1).

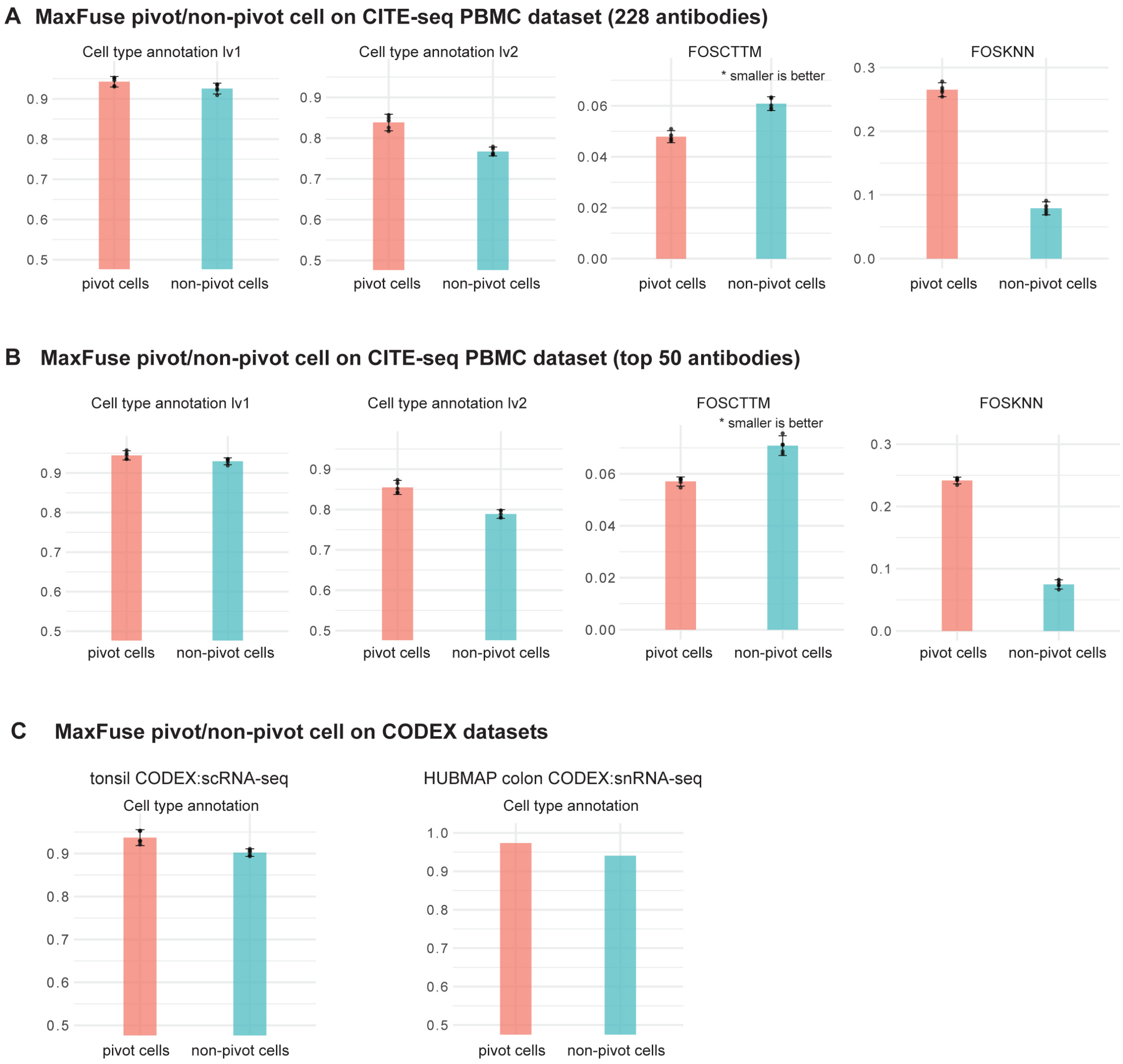

Figure S10: Comparison of matching quality between pivot and non-pivot cells. **(A)** Benchmarking metrics of 'pivot' and 'non-pivot' cells from CITE-seq PBMC data (1) (full 228 antibody panel) ground-truth testing over 5 random batches (10,000 random cells in each batch). The barplot of cell type matching accuracy shows mean value with 95% CI for each group, with raw values from five random samples plotted as dots. **(B)** Benchmarking metrics of 'pivot' and 'non-pivot' cells from CITE-seq PBMC data (1) (top 50 antibodies) ground-truth testing over 5 random batches (10,000 random cells in each batch). The barplot of cell type matching accuracy shows mean value with 95% CI for each group, with raw values from five random samples plotted as dots. **(C)** Cell type annotation matching accuracy of 'pivot' and 'non-pivot' cells from spatial proteomic-related integration. Left: Tonsil CODEX & scRNA-seq (5, 6) integration over 5 random batches (10,000 scRNA-seq cells and 30,000 CODEX cells in each batch). The barplot of cell type matching accuracy shows mean value with 95% CI for each group, with raw values from five random samples plotted as dots. Right: HUBMAP colon CODEX & snRNA-seq (7) integration.

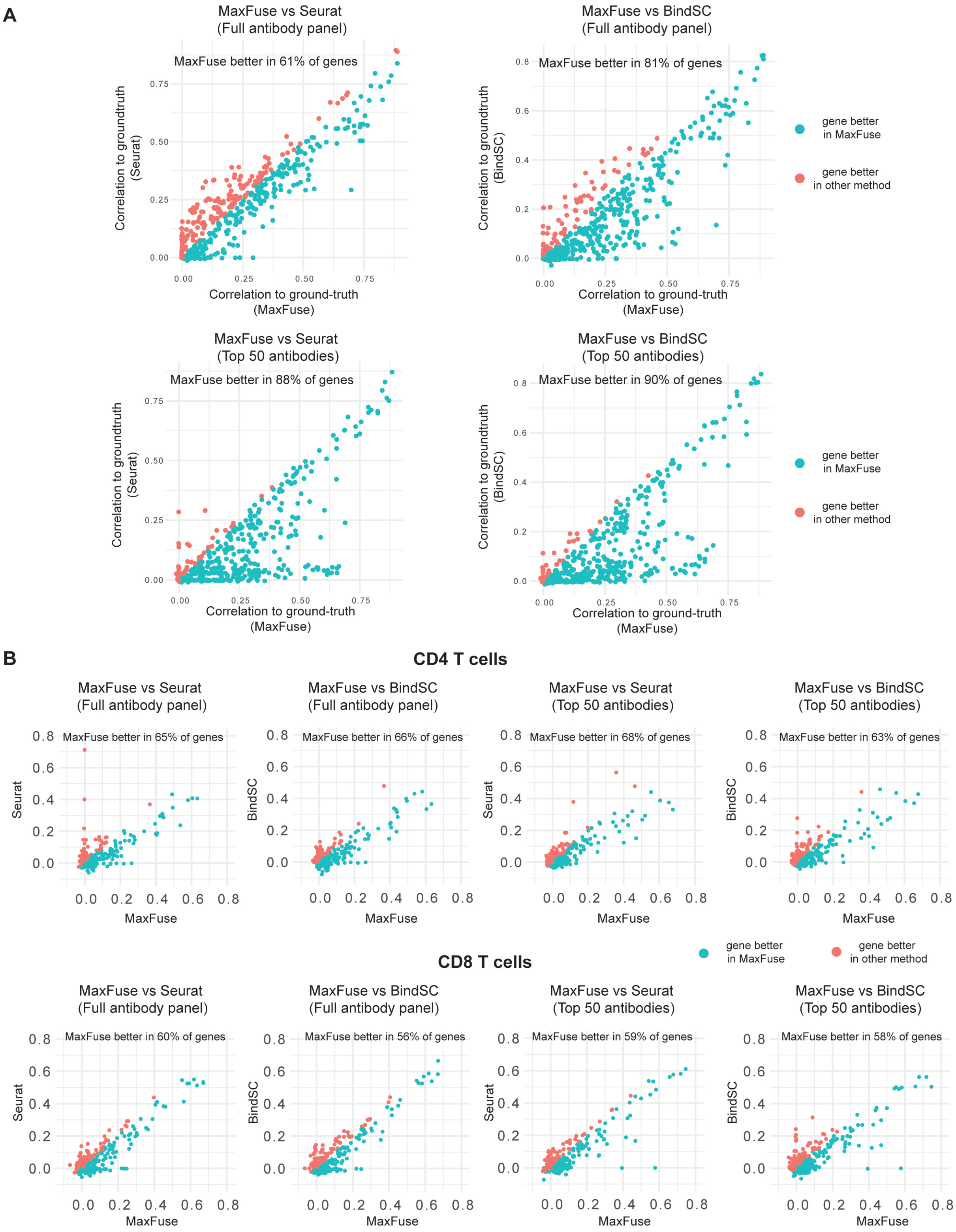

Figure S11: Benchmarking of MaxFuse with other integration methods in terms of prediction accuracy of highly variable genes on ground-truth CITE-seq PBMC data (1). (A) Scatter plots of correlations between predicted gene expression levels of top 500 highly variable genes across all cell types via matching and ground-truth expression levels (Left: Seurat V3 vs. MaxFuse; Right: BindSC vs. MaxFuse). Each dot represents a highly variable gene. Experiment was conducted on 10,000 randomly chosen cells. Correlations between predicted and ground-truth expression levels for each gene were computed over the chosen 10,000 cells. Top row: matching with full antibody panel (228 antibodies). Bottom row: matching with top 50 antibodies. **(B)** Scatter plots of correlations between predicted gene expression levels of top 500 highly variable genes within a level 1 cell type via matching and ground-truth expression levels when the top 50 antibodies were used in integration. All other settings were the same as those in panel (A). Top row: CD4 T cells. Bottom row: CD8 T cells. Left to right within either row: Seurat V3 vs. MaxFuse (full panel, 228 antibodies), BindSC vs. MaxFuse (full panel, 228 antibodies), Seurat V3 vs. MaxFuse (top 50 antibodies), and BindSC vs. MaxFuse (top 50 antibodies)

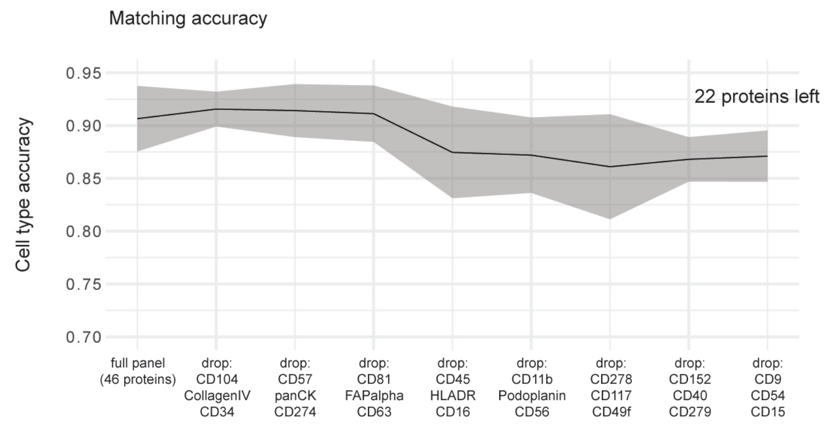

Figure S12: Benchmarking of MaxFuse performance on spatial-omics integration with sequentially reduced CODEX protein panels on human tonsil data (5, 6). Importance of individual protein markers was scored after fitting a random forest model for predicting human-expert cell type annotation. Starting with a full panel of 46 markers, in each round, the three protein markers with lowest importance scores among the remaining markers were dropped and MaxFuse was used to integrate CODEX and scRNA-seq data with the reduced protein panel. Cell type matching accuracy of MaxFuse integration was recorded. A total of 8 rounds of panel reduction experiments were run, each time on 5 random batches with 10,000 scRNA-seq cells and 30,000 CODEX cells, and 22 proteins were left in the last round. Line indicates mean value and shadow indicates 95% CI on both sides.

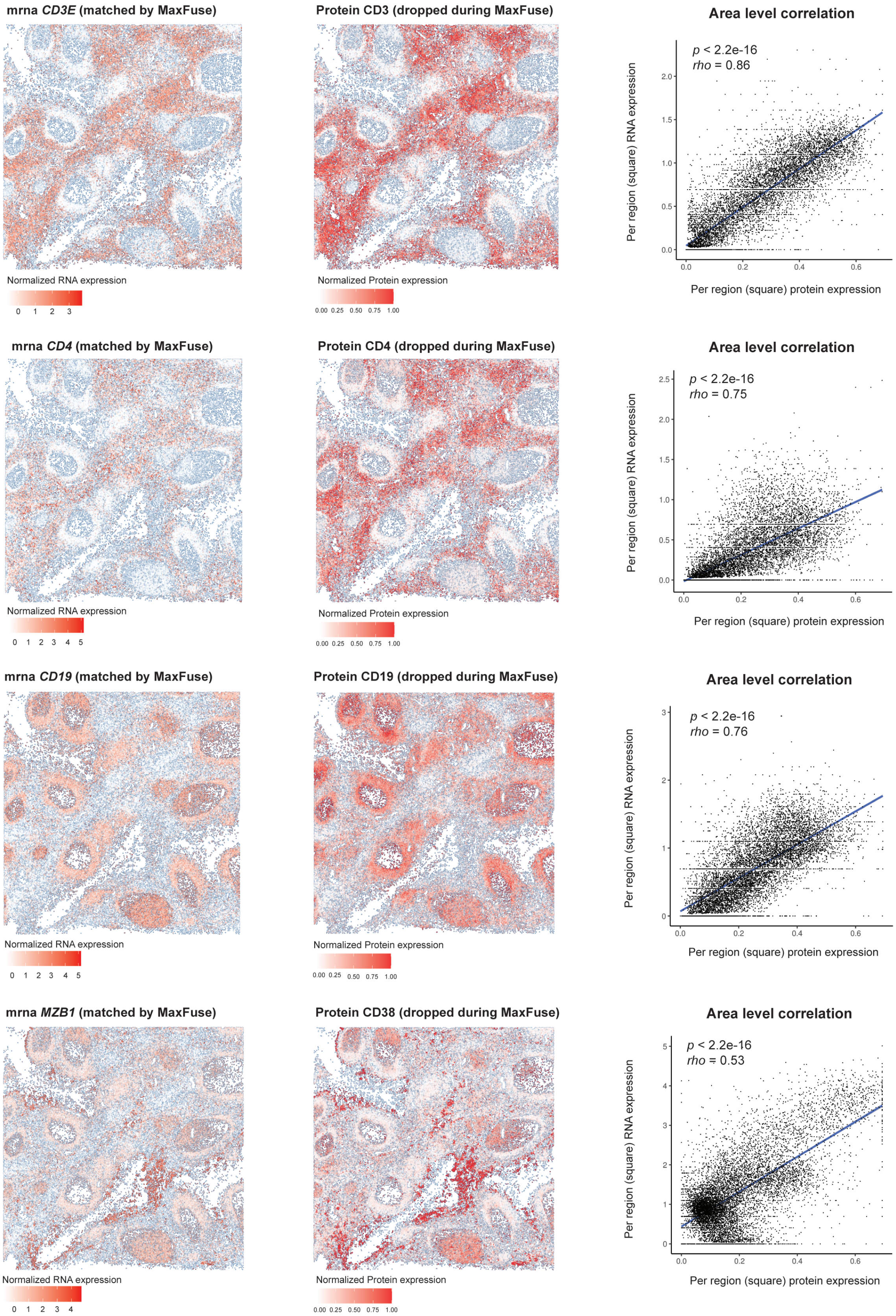

Figure S13: Benchmarking of MaxFuse prediction of spatial distribution of hold-out protein by mRNA expression levels of matched cells on ground-truth human tonsil data (5, 6). Tonsil CODEX & scRNA-seq integration was performed, where in 4 separate cases, one protein was held out. Spatial Spearman correlations (ρ) between ground truth and prediction were then calculated based on aggregated values in each region (See Methods for detail). Left: mRNA expression level spatial distribution of the coding gene for the hold-out protein, based on MaxFuse matched scRNA-seq cells. Middle: spatial distribution of the hold-out protein. Right: scatter plot of mRNA expression level of matched cells vs. ground-truth protein abundance, both values were region-aggregated. P value calculated from a two-sided t-test (`cor.test` from R package 'stats'), blue line indicates a fitted linear regression model (`geom_smooth(lm)` from R package 'ggplot').

Supplementary Methods

A batched version of MaxFuse. Single-cell and spatial datasets can be large. To facilitate fast computation for large datasets, we developed a batched version of MaxFuse.

Batching Fix a desired pair of sample sizes (n_y, n_z) and meta-cell ratios $(N_y/n_y, N_z/n_z)$, we randomly partition the dataset under Y-modality (resp. Z-modality) into disjoint subsets of sizes roughly all equal to N_y (resp. N_z). Denote them as $Y^{[1]}, \ldots, Y^{[b_y]}$ and $Z^{[1]}, \ldots, Z^{[b_z]}$. We then apply the MaxFuse pipeline on each pair of data $\{Y^{[l]}, Z^{[m]}\}$, $1 \leq l \leq b_y$, $1 \leq m \leq b_z$ to get the refined pivots and the propagated matching, as well as their induced single-cell level matched pairs, for that pair of batches.

Stitching After pooling all refined pivots from all batch pairs, we obtain a multiple-to-multiple matching. For each unique cell in Z-modality, we average all its matches in Y-modality, that is, we average matched cells in the modality with a higher SNR. After this step, we get a pair of matrices with rows paired. We then fit CCA on this pair of matrices and get the loading matrices, which are then used to jointly embed the whole datasets. Finally, with the joint embedding of the whole datasets in Y- and Z-modalities, scoring and directional pruning of matching are performed in the same way as in MaxFuse without batching. The current implementation of MaxFuse uses batching by default. We suggest setting n_y (i.e., the parameter `max_outward_size` in MaxFuse package) in between 3,000-10,000 with default value 5,000, and 3-5 for n_z/n_y (i.e., `matching_ratio`) with default value 4. See Extended Data Figure 8 for benchmark results demonstrating the effects of batching on run-time and memory usage of the MaxFuse pipeline and for an example of integrating up to 2 million CODEX cells with batching.

Benchmarking on ground-truth strongly linked modalities.

MaxFuse and specialized ATAC-RNA integration methods in comparison We compared MaxFuse to three methods that specialize in ATAC-RNA integration: scGLUE (8), scJoint (9), and Maestro (10). For MaxFuse, the initial matching used the gene activity scores, while during refined matching the active RNA features and LSI embedding from ATAC were used. For other methods in comparison, we used their default settings. Metrics used for benchmarking were calculated similarly as described in previous sections. The full detail (including preprocessing, implementation, and downstream analysis and evaluation of MaxFuse and other methods specialized in ATAC-RNA integration) was recorded and can be reproduced, with code deposited to `https://github.com/shuxiaoc/maxfuse/tree/main/Archive`.

Multiome scRNA - scATAC-seq human retina dataset analysis. Multiome (scRNA-seq & scATAC-seq) data of human retina cells was retrieved from Wang et al. (11). For input required by MaxFuse: gene activity scores and LSI scores of ATAC cells were calculated by R package `Archr` using the fragment files, while RNA counts were directly extracted. For other methods in comparison, we used their default settings. For benchmarking, a total of 20,000 cells were randomly sampled and used for testing. All cell types were used during integration ("Rod", "OFF cone bipolar", "Mullerglia", "ON cone bipolar", "Rod bipolar", "Cone", "GABA amacrine", "Horizontal", "Glyamacrine", "AII amacrine", "Retinal ganglion cell", "Astrocyte", "Microglia", annotated by Wang et al.). The first 15 components of the embedding vectors produced by all methods were used for benchmarking metric calculation.

10x multiome peripheral blood mononuclear cells dataset analysis. Multiome (scRNA-seq & scATAC-seq) data of human mononuclear peripheral blood cells was retrieved from the 10x public data repository (12). For input required by MaxFuse: gene activity scores and LSI scores of ATAC modality were calculated by R package `Signac`, the latter using the fragment files. RNA counts were directly extracted from the `cellranger` output. Cell-type labels were transferred from CITE-seq PBMC reference (1) using the method in (1).

10x multiome day 18 embryonic mouse brain cells dataset analysis. Multiome (scRNA-seq & scATAC-seq) data of developing mouse brain cells were retrieved from the 10x public data repository (12). For input required by MaxFuse: gene activity scores and LSI scores of ATAC modality were calculated by R package `Signac`, the latter using the fragment files. RNA counts were directly extracted from the `cellranger` output. Cell-type labels were transferred from (13) using the method in (14).

10x multiome developing human cerebral cortex cells dataset analysis. Multiome (scRNA-seq & scATAC-seq) data of developing human cerebral cortex cells were retrieved from Trevino et al. (15). For input required by MaxFuse: gene activity scores and LSI scores of ATAC modality were calculated by R package `Signac` using the fragment files. RNA counts and ATAC peak matrices were extracted from 10x `cellranger` output. The cell-type labels were taken from the original publication.

Runtime comparison in ATAC-RNA integration. We randomly sampled four subsets of the human retina dataset from Wang et al. (11). The subsets have 2,500, 5,000, 10,000, and 20,000 cells, respectively. For each subset, we applied each of the four methods (MaxFuse, scGLUE, scJoint, and Maestro) five times to integrate RNA and ATAC modalities of the cells. We recorded runtimes of all data-method-replication combinations. Methods with GPU option (scJoint and scGLUE) were tested under both

CPU-only and GPU modes. To ensure that we focused on comparing integration runtime, we pre-computed gene activity scores and LSI scores using R package `Archr` for the ATAC modality for MaxFuse, and LSI scores using `scglue.data.lsi` in its own Python package for scGLUE. These computations were not counted toward reported integration runtimes.
MaxFuse, scJoint (CPU-only), and scGLUE (CPU-only) experiments were performed on a MacBook Pro laptop with M1 Max CPU. scJoint (GPU) and scGLUE (GPU) experiments were performed on a Linux workstation with dual Intel i9-10980XE CPUs and dual NVIDIA Quadro RTX 8000 GPUs. Due to M1 silicon incompatibility, Maestro experiments were performed on a Linux workstation with dual Intel i9-10980XE CPUs and dual NVIDIA Quadro RTX 8000 GPUs. The reported Maestro runtimes were calibrated against scJoint runtimes on both computing platforms to ensure fair comparison.

Experimental validation of predicted mRNA spatial pattern. To validate the mRNA spatial patterns of *AICDA* and *CCR6* predicted by MaxFuse cell matching in human tonsil data, we performed RNAscope (Advanced Cell Diagnostics, Newark, CA), following the manual provided by the vendor (with RNAscope Multiplex Fluorescent Reagent Kit v2, Hs-AICDA-C1 and Hs-CCR6-C2 probes). To obtain the images, fresh frozen human tonsil tissue was retrieved from -80°C, fixed by 10% NBF at 4°C for 1 hr, and washed with 1 × PBS. Subsequently, the slide was dehydrated by a sequential wash of 50%, 70%, 100%, and 100% EtOH, each for 5 mins. Afterwards, the slide was treated with hydrogen peroxide (3%) for 1 min at RT, washed by ddH2O, then followed by tissue digestion with RNAscope Protease III for 1 min at RT. Probes were then hybridized at 40°C for 2 hrs, followed by 2 times washing with 1 × RNAscope wash buffer at RT. Next, the amplification process was performed in sequal as: 1) Amp1, 30 mins at 40°C, 2) Amp2, 30 mins at 40°C, and 3) Amp3, 15 mins at 40°C. After each amplification step, the slide was washed 2 times with 1 × RNAscope wash buffer at RT. The slide was then stained with HRP-C1, 15 mins at 40°C, washed 2 times with 1 × RNAscope wash buffer at RT, followed by TSA signal deposition using Cy3-TSA (1:700 dilution in TSA buffer, 30 mins at 40°C). The process for C2 was similar but with Cy5-TSA. Finally, the slide was stained with DAPI, and imaged by a 20 × objective using a Keyence BZ-X710 fluorescent microscope.

Additional MaxFuse benchmark results. To gain further insights into the MaxFuse pipeline, we conducted additional benchmark tests in a variety of settings.

Matching accuracy of pivot vs. non-pivot cells To delineate the performance difference between pivot cells and non-pivot cells in MaxFuse integration, we evaluated different metrics comparing pivot cells and non-pivot cells across four different scenarios: 1) ground-truth CITE-seq PBMC data (1) with full 228 antibody panel; 2) the same CITE-seq PBMC data (1) with top 50 antibodies; 3) tonsil CODEX & scRNA-seq integration (5, 6); 4) HUBMAP colon CODEX & snRNA-seq integration (7). For scenario 1) and 2), cell type matching accuracy (both level 1 and level 2), FOSCTTM, and FOSKNN were computed separately for pivot and non-pivot cells. For scenario 3) and 4), only cell type matching accuracy was computed as these are datasets without ground-truth knowledge on matching between RNA and CODEX cells.

Effect of fuzzy smoothing Fuzzy smoothing is one of the key innovations that enable MaxFuse to integrate weakly linked modalities, especially when a spatial proteomic dataset serves as one modality. To evaluate the advantage of using fuzzy smoothing, we created four scenarios for comparison: 1) with smoothing ($w = 0.3$), i.e., the setting used in the analyses reported in Figure 4 and 5; 2) no smoothing ($w = 1$); 3) construct random NN-graph and run smoothing ($w = 0.3$); 4) construct random NN-graph and run smoothing ($w = 0.1$). The same w was used in both initialization and refinement. Random NN-graph construction: NN-graph was first constructed by the function `pp.neighbors` in Python package `scanpy`, with Leiden clustering performed by function `tl.leiden` in `scanpy` with `resolution = 2`. Leiden cluster labels were randomly shuffled and then a random label was assigned to each cell. In scenario 3) and 4), the graph neighborhood of a cell consisted of all cells sharing its random cluster label, and smoothing was performed over these random neighborhoods of cells. Two separate cases involving spatial proteomic datasets were tested: 1) tonsil CODEX & scRNA-seq integration (5, 6), and 2) HUBMAP colon CODEX & snRNA-seq integration (7).

Cell population mismatch test We benchmarked performance of MaxFuse with mismatched cell populations in two modalities on ground-truth CITE-seq PBMC data (1). In each experiment, we dropped all cells in a chosen level 2 cell type within the protein modality, performed MaxFuse integration of the full RNA dataset and the reduced protein dataset with and without filtering, and recorded four evaluation metrics: percentage of RNA cells (out of all RNA cells) from the dropped cell type that were matched to protein cells without filtering used in MaxFuse, the same quantity with filtering used, proportion of matched pairs with the same level 2 cell type annotation without filtering used in MaxFuse, and the same quantity with filtering used. For reference, we also recorded the mismatch proportion which is the percentage of RNA cells belonging to the dropped cell type. We tested on three different level 2 cell types: "CD16 Mono", "CD8 TEM", and "CD4 Naive". For each cell type, we performed repetitions over 5 random batches of 10,000 cells.

Benchmarking of MaxFuse gene imputation accuracy on ground-truth CITE-seq PBMC data MaxFuse integration was performed on ground-truth CITE-seq PBMC data (1) (10,000 randomly sampled cells), with either the full antibody panel with 228 antibodies or a reduced panel with top 50 antibodies. For both antibody panel sizes, gene imputation accuracy on three

different sets of cells was considered: 1) all cells across all cell types, 2) all cells with level 1 annotation "CD4 T", and 3) all cells with level 1 annotation "CD8 T". Within each cell set, the top 500 highly variable genes were selected by the function `pp.highly_variable_genes` in Python package `scanpy`. Within each cell set, for each highly variable gene, Pearson correlation across cells was calculated based on each protein modality cell's ground-truth RNA expression and its matched RNA modality cell's RNA expression. The performance of MaxFuse was benchmarked against Seurat V3 and BindSC on the same cells and the same highly variable genes. For visualization purpose, genes with Pearson correlation values 0 produced by both methods in comparison were removed from scatter plots in Supplementary Figure 18.

Sequential antibody panel reduction test for spatial-omics matching To evaluate the performance of MaxFuse with smaller antibody panel sizes, we conducted a sequential antibody panel reduction test on MaxFuse integration of human tonsil CODEX & scRNA-seq data (5, 6). A random forest model was trained to predict cell types from expressions of all 46 protein markers, with the function `randomForest` in R package `randomForest` and default parameters. Then, a permutation test was performed to determine the importance of individual protein markers in the model, using function `varImp` with default parameters in R package `caret`, which resulted in an importance score for each protein. We then sequentially dropped protein markers based on their importance scores, where progressively in each round the 3 least important ones among the remaining proteins were dropped, and the matching accuracy of MaxFuse with the reduced panel was assessed. We performed 8 rounds of panel reduction and the panel size in the last round was 22. In each round, we repeated the experiment on 5 random batches with 10,000 scRNA-seq cells and 30,000 CODEX cells.

Prediction of hold-out protein spatial distribution in spatial-omics matching To evaluate whether the spatial pattern of a left-out protein in a spatial proteomic dataset can be predicted by that of the corresponding mRNA in MaxFuse-matched cells, we performed hold-out experiments on tonsil CODEX & scRNA-seq data (5, 6). When integrating human tonsil CODEX & scRNA-seq data, we held out each of CD3, CD4, CD19, and CD38 proteins from CODEX data in four separate runs, and in each run evaluated whether the spatial pattern of the corresponding mRNA obtained from MaxFuse-matched scRNA-seq cells could predict local expression levels of the hold-out protein. We measured prediction accuracy by an area level Spearman's rho correlation. When calculating this metric for a protein-mRNA pair, the entire CODEX image was divided into 10,000 identical squares (100 rows by 100 columns, with area of each square around $35 \times 35 \mu m^2$), and the Spearman's rho correlation across areas was calculated based on cumulative protein expressions and cumulative expressions of the corresponding mRNA by MaxFuse integration in individual squares.

Benchmarking against automated cell annotation methods designed for spatial proteomic datasets In order to compare MaxFuse label transfer performance to other state-of-the-art automated cell annotation methods designed for spatial proteomic datasets, we implemented CELESTA (11) and Astir (16) on the human tonsil CODEX data, and compared cell annotation accuracy assuming the original human-expert annotation is ground truth. For MaxFuse, the matched scRNA-seq cell type for each CODEX cell was treated as annotation. For CELESTA, the input consisted of arcsin-transformed raw CODEX expression values, and the parameters were set as `high_marker_threshold = 0.9`, `low_marker_threshold = 0.4` for function `FilterCells`, and `max_iteration = 10`, `cell_change_threshold = 0.01` for function `AssignCells`. For Astir, the input consisted of Z-normed raw CODEX expression values, and the default parameters were used. Cells annotated as 'dirt' by Astir were relabeled to 'Unknown' in downstream analysis.

Supplementary Tables

Name of dataset	Type of data	Pivot perc (RNA)	Pivot perc (Protein)
CITE-seq-PBMC (228 antibodies)	Protein – RNA; non-spatial	69%	28%
CITE-seq-PBMC (50 antibodies)	Protein – RNA; non-spatial	70%	29%
tonsil-CODEX-scRNA-seq	Protein – RNA; spatial	51%	7%
hubmap-CODEX-snRNA-seq	Protein – RNA; spatial	79%	23%

Table 1. Percentages of cells in different modalities in pivots. The difference of percentages in RNA and Protein modalities in CITE-seq-PBMC examples (first two rows) is due to meta-cell construction within RNA modality. When a meta-cell entered a pivot, all its member cells were included in the calculation of pivot percentage. In these two examples, each meta-cell consisted of two cells on average, while the number of cells going into each meta-cell varied. This resulted in a roughly 2:1 ratio in the pivot percentages in the first two rows.

Name of dataset	Type of data	'meta cell' size
CITE-seq-PBMC	Protein – RNA; non-spatial	2
CITE-seq-BMC	Protein – RNA; non-spatial	2
ASAPseq-BMC	Protein – ATAC; non-spatial	2
teaseq-PBMC	Protein – ATAC; non-spatial	2
tonsil-CODEX-scRNA-seq	Protein – RNA; spatial	3
hubmap-CODEX-scRNA-seq	Protein – RNA; spatial	3
hubmap-scRNA-seq-scATAC-seq	RNA – ATAC; non-spatial	3

Table 2. Summary of meta-cell size (i.e., average number of single cells used in constructing a meta-cell = ratio of single cell count over meta-cell count) used for each analyzed dataset. For all datasets involving protein and RNA, meta-cell construction is restricted to the RNA modality. For all datasets involving protein and ATAC, meta-cell construction is restricted to the ATAC modality. For RNA-ATAC integration, meta-cell construction is restricted to the RNA modality.

Name of dataset	Type of data	'w' used (initial/refine)
CITE-seq-PBMC	Protein – RNA; non-spatial	0.7/0.7
CITE-seq-BMC	Protein – RNA; non-spatial	0.7/0.7
ASAPseq-BMC	Protein – ATAC; non-spatial	0.7/0.7
teaseq-PBMC	Protein – ATAC; non-spatial	0.7/0.7
tonsil-CODEX-scRNA-seq	Protein – RNA; spatial	0.3/0.3
hubmap-CODEX-scRNA-seq	Protein – RNA; spatial	0.3/0.3
hubmap-scRNA-seq-scATAC-seq	RNA – ATAC; non-spatial	0.7/0.7

Table 3. Summary of fuzzy-smoothing weights used in initialization and in refinement for the analyzed datasets. Lower value of the weight corresponds to more smoothing.

Name of dataset	Type of data	'α' used (pivot/full)
CITE-seq-PBMC	Protein – RNA; non-spatial	0.3/0
CITE-seq-BMC	Protein – RNA; non-spatial	0.3/0
ASAPseq-BMC	Protein – ATAC; non-spatial	0.3/0
teaseq-PBMC	Protein – ATAC; non-spatial	0.2/0
tonsil-CODEX-scRNA-seq	Protein – RNA; spatial; benchmark	0.5/0.3
tonsil-CODEX-scRNA-seq	Protein – RNA; spatial; analysis	0.5/0.3
hubmap-CODEX-scRNA-seq	Protein – RNA; spatial	0.3/0.3
hubmap-scRNA-seq-scATAC-seq	RNA – ATAC; non-spatial	0/0

Table 4. Summary of filtering percentage used on pivot and full matching for each analyzed dataset. Lower value of filtering percentage α corresponds to less filtering.

Dropped cell type	Matched percentage (w/o filtering)	Matched percentage (w/ filtering)	Mismatch proportion	lv2 accuracy (w/o filtering)	lv2 accuracy (w filtering)
CD16 Monocyte	1.74%	1.00%	3.98%	78.7%	82.9%
CD8 TEM	1.84%	2.22%	7.29%	78.4%	81.8%
CD4 Naive	6.79%	5.56%	12.02%	73.4%	76.9%

Table 5. Benchmarking of robustness of MaxFuse with mismatched cell populations in two modalities on ground-truth CITE-seq PBMC data (1). In three experiments, all cells with level 2 annotation "CD16 Mono", "CD8 TEM", and "CD4 Naive" were removed in the protein modality, respectively. Matched percentage indicates the percentage of cells matched to deleted cell types (the smaller the better, indicating less erroneous matching to non-exist cell types) with or without filtering. Mismatch proportion indicates percentage of RNA cells belonging to the dropped cell type. lv2 accuracy is the overall matching accuracy for all cells measured in level 2 annotation agreement of matched pairs, with or without filtering. All results reported were averaged over 5 random batches of 10,000 cells. For all results involving filtering, filtering proportion α = 0.3 was used for both pivot and full matching. Level 2 annotations of cells were taken from (1).

Reference

1. Yuhan Hao, Stephanie Hao, Erica Andersen-Nissen, William M Mauck III, Shiwei Zheng, Andrew Butler, Maddie J Lee, Aaron J Wilk, Charlotte Darby, Michael Zager, et al. Integrated analysis of multimodal single-cell data. *Cell*, 184(13):3573–3587, 2021.
2. Eleni P Mimitou, Caleb A Lareau, Kelvin Y Chen, Andre L Zorzetto-Fernandes, Yuhan Hao, Yusuke Takeshima, Wendy Luo, Tse-Shun Huang, Bertrand Z Yeung, Efthymia Papalexi, et al. Scalable, multimodal profiling of chromatin accessibility, gene expression and protein levels in single cells. *Nature biotechnology*, 39(10):1246–1258, 2021.
3. Sergio Triana, Dominik Vonficht, Lea Jopp-Saile, Simon Raffel, Raphael Lutz, Daniel Leonce, Magdalena Antes, Pablo Hernández-Malmierca, Diana Ordoñez-Rueda, Beáta Ramasz, et al. Single-cell proteo-genomic reference maps of the hematopoietic system enable the purification and massive profiling of precisely defined cell states. *Nature immunology*, 22(12):1577–1589, 2021.
4. Elliott Swanson, Cara Lord, Julian Reading, Alexander T Heubeck, Palak C Genge, Zachary Thomson, Morgan DA Weiss, Xiao-jun Li, Adam K Savage, Richard R Green, et al. Simultaneous trimodal single-cell measurement of transcripts, epitopes, and chromatin accessibility using tea-seq. *Elife*, 10:e63632, 2021.
5. Julia Kennedy-Darling, Salil S Bhate, John W Hickey, Sarah Black, Graham L Barlow, Gustavo Vazquez, Vishal G Venkataraaman, Nikolay Samusik, Yury Goltsev, Christian M Schürch, et al. Highly multiplexed tissue imaging using repeated oligonucleotide exchange reaction. *European Journal of Immunology*, 51(5):1262–1277, 2021.
6. Hamish W King, Kristen L Wells, Zohar Shipony, Arwa S Kathiria, Lisa E Wagar, Caleb Lareau, Nara Orban, Robson Capasso, Mark M Davis, Lars M Steinmetz, et al. Integrated single-cell transcriptomics and epigenomics reveals strong germinal center–associated etiology of autoimmune risk loci. *Science Immunology*, 6(64):eabh3768, 2021.
7. John W Hickey, Winston R Becker, Stephanie A Nevins, Aaron Horning, Almudena Espin Perez, Roxanne Chiu, Derek C Chen, Daniel Cotter, Edward D Esplin, Annika K Weimer, et al. High resolution single cell maps reveals distinct cell organization and function across different regions of the human intestine. *bioRxiv*, 2021.
8. Zhi-Jie Cao and Ge Gao. Multi-omics single-cell data integration and regulatory inference with graph-linked embedding. *Nature Biotechnology*, pages 1–9, 2022.
9. Yingxin Lin, Tung-Yu Wu, Sheng Wan, Jean YH Yang, Wing H Wong, and YX Wang. scjoint integrates atlas-scale single-cell rna-seq and atac-seq data with transfer learning. *Nature Biotechnology*, 40(5):703–710, 2022.
10. Chenfei Wang, Dongqing Sun, Xin Huang, Changxin Wan, Ziyi Li, Ya Han, Qian Qin, Jingyu Fan, Xintao Qiu, Yingtian Xie, et al. Integrative analyses of single-cell transcriptome and regulome using maestro. *Genome biology*, 21(1):1–28, 2020.
11. Sean K Wang, Surag Nair, Rui Li, Katerina Kraft, Anusri Pampari, Aman Patel, Joyce B Kang, Christy Luong, Anshul Kundaje, and Howard Y Chang. Single-cell multiome of the human retina and deep learning nominate causal variants in complex eye diseases. *bioRxiv*, 2022.
12. 10X Genomics. 10x genomics datasets, 2022.
13. Gioele La Manno, Kimberly Siletti, Alessandro Furlan, Daniel Gyllborg, Elin Vinsland, Alejandro Mossi Albiach, Christoffer Mattsson Langseth, Irina Khven, Alex R Lederer, Lisa M Dratva, et al. Molecular architecture of the developing mouse brain. *Nature*, 596(7870):92–96, 2021.
14. Mo Huang, Zhaojun Zhang, and Nancy R Zhang. Dimension reduction and denoising of single-cell rna sequencing data in the presence of observed confounding variables. *bioRxiv*, 2020.
15. Alexandro E Trevino, Fabian Müller, Jimena Andersen, Laksshman Sundaram, Arwa Kathiria, Anna Shcherbina, Kyle Farh, Howard Y Chang, Anca M Pașca, Anshul Kundaje, et al. Chromatin and gene-regulatory dynamics of the developing human cerebral cortex at single-cell resolution. *Cell*, 184(19):5053–5069, 2021.
16. Michael J Geuenich, Jinyu Hou, Sunyun Lee, Shanza Ayub, Hartland W Jackson, and Kieran R Campbell. Automated assignment of cell identity from single-cell multiplexed imaging and proteomic data. *Cell Systems*, 12(12):1173–1186, 2021.
