# Supplementary material for: Integration of spatial and single-cell data across modalities with weakly linked features
Source: Nat Biotechnol. 2023 Sep 7;42(7):1096–106. doi: 10.1038/s41587-023-01935-0 (PMC11638971; doi:10.1038/s41587-023-01935-0)
Supplement: Supplementary file 2 — Reporting Summary [file 41587_2023_1935_MOESM2_ESM.pdf]

nature portfolio

Corresponding author(s): Garry P. Nolan, Nancy R. Zhang, Zongming Ma

Last updated by author(s): Jul 25, 2023

Reporting Summary

Nature Portfolio wishes to improve the reproducibility of the work that we publish. This form provides structure for consistency and transparency in reporting. For further information on Nature Portfolio policies, see our Editorial Policies and the Editorial Policy Checklist.

Statistics

For all statistical analyses, confirm that the following items are present in the figure legend, table legend, main text, or Methods section.

n/a	Confirmed	
☐	☒	The exact sample size (*n*) for each experimental group/condition, given as a discrete number and unit of measurement
☐	☒	A statement on whether measurements were taken from distinct samples or whether the same sample was measured repeatedly
☐	☒	The statistical test(s) used AND whether they are one- or two-sided<br>*Only common tests should be described solely by name; describe more complex techniques in the Methods section.*
☒	☐	A description of all covariates tested
☒	☐	A description of any assumptions or corrections, such as tests of normality and adjustment for multiple comparisons
☐	☒	A full description of the statistical parameters including central tendency (e.g. means) or other basic estimates (e.g. regression coefficient) AND variation (e.g. standard deviation) or associated estimates of uncertainty (e.g. confidence intervals)
☐	☒	For null hypothesis testing, the test statistic (e.g. *F*, *t*, *r*) with confidence intervals, effect sizes, degrees of freedom and *P* value noted<br>*Give P values as exact values whenever suitable.*
☒	☐	For Bayesian analysis, information on the choice of priors and Markov chain Monte Carlo settings
☒	☐	For hierarchical and complex designs, identification of the appropriate level for tests and full reporting of outcomes
☐	☒	Estimates of effect sizes (e.g. Cohen's *d*, Pearson's *r*), indicating how they were calculated

Our web collection on statistics for biologists contains articles on many of the points above.

Software and code

Policy information about availability of computer code

Data collection

All data used in this manuscript manuscript are publicly available and no software were used to collect the data.

Data analysis

All downstream analysis scripts used in this manuscript, including information about all the open source packages (with R=3.6.3, Python=3.8.15) can be accessed under: https://github.com/shuxiaoc/maxfuse/tree/main/Archive; Due to the space limit packages will not be shown in this report. For benchmarking against other methods for integration/matching performance: Seurat (4.1.1 for SeuratV3 matching), Liger (1.0.0), Harmony (0.1.0), BindSC (1.0.0), scGLUE (0.3.0), scJoint (1.0.0), Maestro (1.5.0).

MaxFuse 0.0.0 (development version for the manuscript preparation):
https://github.com/shuxiaoc/maxfuse/tree/main/Archive/MaxFuse_devo/09302022V

MaxFuse 0.0.1 (for publication):
Detailed Python packages used in the MaxFuse software package:

anndata	0.8.0	pypi_0	pypi
appnope	0.1.2	py38hecd8cb5_1001	anaconda
argon2-cffi	21.3.0	pyhd3eb1b0_0	anaconda
argon2-cffi-bindings	21.2.0	py38hca72f7f_0	anaconda
asttokens	2.0.5	pyhd3eb1b0_0	anaconda
attrs	21.4.0	pyhd3eb1b0_0	anaconda
backcall	0.2.0	pyhd3eb1b0_0	anaconda
beautifulsoup4	4.11.1	py38hecd8cb5_0	anaconda
bleach	4.1.0	pyhd3eb1b0_0	anaconda

bzip2	1.0.8	h0d85af4_4	conda-forge
ca-certificates	2022.4.26	hecd8cb5_0	anaconda
certifi	2022.12.7	pypi_0	pypi
cffi	1.15.0	py38hc55c11b_1	anaconda
charset-normalizer	3.0.1	pypi_0	pypi
contourpy	1.0.7	pypi_0	pypi
cycler	0.11.0	pypi_0	pypi
dbus	1.13.18	h18a8e69_0	anaconda
debugpy	1.5.1	py38he9d5cce_0	anaconda
decorator	5.1.1	pyhd3eb1b0_0	anaconda
defusedxml	0.7.1	pyhd3eb1b0_0	anaconda
entrypoints	0.4	py38hecd8cb5_0	anaconda
executing	0.8.3	pyhd3eb1b0_0	anaconda
expat	2.4.4	he9d5cce_0	anaconda
fonttools	4.38.0	pypi_0	pypi
gettext	0.21.0	h7535e17_0	anaconda
glib	2.56.2	hd9629dc_0	anaconda
h5py	3.8.0	pypi_0	pypi
icu	58.2	h0a44026_3	anaconda
idna	3.4	pypi_0	pypi
igraph	0.10.3	pypi_0	pypi
importlib-metadata	6.0.0	pypi_0	pypi
importlib_resources	5.2.0	pyhd3eb1b0_1	anaconda
ipykernel	6.9.1	py38hecd8cb5_0	anaconda
ipython	8.3.0	py38hecd8cb5_0	anaconda
ipython_genutils	0.2.0	pyhd3eb1b0_1	anaconda
ipywidgets	7.6.5	pyhd3eb1b0_1	anaconda
jedi	0.18.1	py38hecd8cb5_1	anaconda
jinja2	3.0.3	pyhd3eb1b0_0	anaconda
joblib	1.2.0	pypi_0	pypi
jpeg	9e	hca72f7f_0	anaconda
jsonschema	4.4.0	py38hecd8cb5_0	anaconda
jupyter	1.0.0	py38_7	anaconda
jupyter_client	7.2.2	py38hecd8cb5_0	anaconda
jupyter_console	6.4.3	pyhd3eb1b0_0	anaconda
jupyter_core	4.10.0	py38hecd8cb5_0	anaconda
jupyterlab_pygments	0.1.2	py_0	anaconda
jupyterlab_widgets	1.0.0	pyhd3eb1b0_1	anaconda
kiwisolver	1.4.4	pypi_0	pypi
leidenalg	0.9.1	pypi_0	pypi
libcxx	12.0.0	h2f01273_0	anaconda
libffi	3.4.2	h0d85af4_5	conda-forge
libiconv	1.16	hca72f7f_2	anaconda
libpng	1.6.37	ha441bb4_0	anaconda
libsodium	1.0.18	h1de35cc_0	anaconda
libsqlite	3.40.0	ha978bb4_0	conda-forge
libxml2	2.9.14	hbf8cd5e_0	anaconda
libzlib	1.2.13	hfd90126_4	conda-forge
llvm-openmp	12.0.0	h0dcd299_1	anaconda
llvmlite	0.39.1	pypi_0	pypi
markupsafe	2.1.1	py38hca72f7f_0	anaconda
matplotlib	3.6.3	pypi_0	pypi
matplotlib-inline	0.1.2	pyhd3eb1b0_2	anaconda
maxfuse	0.0.1	pypi_0	pypi
mistune	0.8.4	py38h1de35cc_1001	anaconda
natsort	8.2.0	pypi_0	pypi
nbclient	0.5.13	py38hecd8cb5_0	anaconda
nbconvert	6.4.4	py38hecd8cb5_0	anaconda
nbformat	5.3.0	py38hecd8cb5_0	anaconda
ncurses	6.3	h96cf925_1	conda-forge
nest-asyncio	1.5.5	py38hecd8cb5_0	anaconda
networkx	3.0	pypi_0	pypi
notebook	6.4.11	py38hecd8cb5_0	anaconda
numba	0.56.4	pypi_0	pypi
numpy	1.23.5	pypi_0	pypi
openssl	3.0.7	hfd90126_1	conda-forge
packaging	23.0	pypi_0	pypi
pandas	1.5.3	pypi_0	pypi
pandocfilters	1.5.0	pyhd3eb1b0_0	anaconda
parso	0.8.3	pyhd3eb1b0_0	anaconda
patsy	0.5.3	pypi_0	pypi
pcre	8.45	h23ab428_0	anaconda
pexpect	4.8.0	pyhd3eb1b0_3	anaconda
pickleshare	0.7.5	pyhd3eb1b0_1003	anaconda
pillow	9.4.0	pypi_0	pypi
pip	22.3.1	pyhd8ed1ab_0	conda-forge

prometheus_client 0.13.1 pyhd3eb1b0_0 anaconda
prompt-toolkit 3.0.20 pyhd3eb1b0_0 anaconda
prompt_toolkit 3.0.20 hd3eb1b0_0 anaconda
ptyprocess 0.7.0 pyhd3eb1b0_2 anaconda
pure_eval 0.2.2 pyhd3eb1b0_0 anaconda
pycparser 2.21 pyhd3eb1b0_0 anaconda
pygments 2.11.2 pyhd3eb1b0_0 anaconda
pynndescent 0.5.8 pypi_0 pypi
pyparsing 3.0.9 pypi_0 pypi
pyqt 5.9.2 py38h655552a_2 anaconda
pyrsistent 0.18.0 py38hca72f7f_0 anaconda
python 3.8.15 hf9b03c3_0_cpython conda-forge
python-dateutil 2.8.2 pyhd3eb1b0_0 anaconda
python-fastjsonschema 2.15.1 pyhd3eb1b0_0 anaconda
pytz 2022.7.1 pypi_0 pypi
pyzmq 22.3.0 py38he9d5cce_2 anaconda
qt 5.9.7 h468cd18_1 anaconda
qtconsole 5.3.0 pyhd3eb1b0_0 anaconda
qtpy 2.0.1 pyhd3eb1b0_0 anaconda
readline 8.1.2 h3899abd_0 conda-forge
requests 2.28.2 pypi_0 pypi
scanpy 1.9.1 pypi_0 pypi
scikit-learn 1.2.0 pypi_0 pypi
scipy 1.10.0 pypi_0 pypi
seaborn 0.12.2 pypi_0 pypi
send2trash 1.8.0 pyhd3eb1b0_1 anaconda
session-info 1.0.0 pypi_0 pypi
setuptools 66.1.1 pyhd8ed1ab_0 conda-forge
sip 4.19.8 py38h0a44026_0 anaconda
six 1.16.0 pyhd3eb1b0_1 anaconda
soupsieve 2.3.1 pyhd3eb1b0_0 anaconda
sqlite 3.38.5 h707629a_0 anaconda
stack_data 0.2.0 pyhd3eb1b0_0 anaconda
statsmodels 0.13.5 pypi_0 pypi
stdlib-list 0.8.0 pypi_0 pypi
terminado 0.13.1 py38hecd8cb5_0 anaconda
testpath 0.6.0 py38hecd8cb5_0 anaconda
texttable 1.6.7 pypi_0 pypi
threadpoolctl 3.1.0 pypi_0 pypi
tk 8.6.12 h5dbffcc_0 conda-forge
tornado 6.1 py38h9ed2024_0 anaconda
tqdm 4.64.1 pypi_0 pypi
traitlets 5.1.1 pyhd3eb1b0_0 anaconda
typing-extensions 4.1.1 hd3eb1b0_0 anaconda
typing_extensions 4.1.1 pyh06a4308_0 anaconda
umap-learn 0.5.3 pypi_0 pypi
urllib3 1.26.14 pypi_0 pypi
wcwidth 0.2.5 pyhd3eb1b0_0 anaconda
webencodings 0.5.1 py38_1 anaconda
wheel 0.38.4 pyhd8ed1ab_0 conda-forge
widgetsnbextension 3.5.2 py38hecd8cb5_0 anaconda
xz 5.2.6 h775f41a_0 conda-forge
zeromq 4.3.4 h23ab428_0 anaconda
zipp 3.11.0 pypi_0 pypi
zlib 1.2.13 hfd90126_4 conda-forge

############# packages utilized in R for downstream analysis

ggplot2 3.4.1
ggsci 2.9
Seurat 3.2.3 or 4.1.1
tidyverse 2.0.0
SeuratData 0.2.1
Matrix 1.5-3
reshape2 1.4.4
dplyr 1.1.1
data.table 1.14.4
harmony 0.1.0
ArchR 1.0.1
chromVAR 1.8.0
chromVARmotifs 0.2.0

For manuscripts utilizing custom algorithms or software that are central to the research but not yet described in published literature, software must be made available to editors and reviewers. We strongly encourage code deposition in a community repository (e.g. GitHub). See the Nature Portfolio guidelines for submitting code & software for further information.

Data

Policy information about availability of data

All manuscripts must include a data availability statement. This statement should provide the following information, where applicable:

- Accession codes, unique identifiers, or web links for publicly available datasets
- A description of any restrictions on data availability
- For clinical datasets or third party data, please ensure that the statement adheres to our policy

All data used in this manuscript are publicly available and no new experiment was performed. The links are listed here:

CITE-seq PBMC from Hao et al.: https://atlas.fredhutch.org/data/nygc/multimodal/pbmc_multimodal.h5seurat (file: 'pbmc_multimodal.h5seurat')
CITE-seq BMC from Hao et al.: https://satijalab.org/seurat/articles/multimodal_reference_mapping.html (file: 'bmcite' with 'SeuratData')
Ab-seq BMC from Triana et al.: https://figshare.com/articles/dataset/
Expression_of_97_surface_markers_and_RNA_transcriptome_wide_in_13165_cells_from_a_healthy_young_bone_marrow_donor/13397987
TEA-seq PBMC from Swanson et al.: ncbi.nlm.nih.gov/geo/query/acc.cgi?acc=GSM4949911
ASAP-seq PBMC from Mimitou et al.: https://www.ncbi.nlm.nih.gov/geo/query/acc.cgi?acc=GSE156473 (GSM4732109 and GSM4732110)
CODEX tonsil from Kennedy et al.: https://onlinelibrary.wiley.com/doi/10.1002/eji.202048891 (will upload raw/processed images/dataframes)
scRNA-seq tonsil from King et al.: https://www.ncbi.nlm.nih.gov/geo/query/acc.cgi?acc=GSE165860 (tonsil 1a, 1b, 2a, 2b, 3a, 3b)
HUBMAP data from Hickey et al.: https://portal.hubmapconsortium.org/search?mapped_data_types[0]=CODEX&mapped_data_types[1]=snRNA-seq%20%2810x%20Genomics%20v3%29&origin_sample.mapped_organ[0]=Small%20Intestine&origin_sample.mapped_organ[1]=Large%20Intestine&entity_type[0]=Dataset&p=1 (will upload raw/processed images/dataframes)
Multiome (scRNA-seq & scATAC-seq) retina from Wang et al.: https://www.ncbi.nlm.nih.gov/geo/query/acc.cgi?acc=GSM5866073
Multiome (scRNA-seq & scATAC-seq) PBMC from 10x genomics datasets: https://www.10xgenomics.com/resources/datasets (PBMC from a Healthy Donor - Granulocytes Removed Through Cell Sorting (10k))
Multiome (scRNA-seq & scATAC-seq) E18 from 10x genomics datasets: https://www.10xgenomics.com/resources/datasets (Fresh Embryonic E18 Mouse Brain (5k))
Multiome (scRNA-seq & scATAC-seq) cerebral cortex from Trevino et al.: https://www.ncbi.nlm.nih.gov/geo/query/acc.cgi?acc=GSE162170 (multiome samples)

Human research participants

Policy information about studies involving human research participants and Sex and Gender in Research.

Reporting on sex and gender	Not applicable for this study.
Population characteristics	Not applicable for this study.
Recruitment	No additional human patient were recruited for this study therefore not applicable.
Ethics oversight	No additional human sample were collected specifically for this study therefore not applicable.

Note that full information on the approval of the study protocol must also be provided in the manuscript.

Field-specific reporting

Please select the one below that is the best fit for your research. If you are not sure, read the appropriate sections before making your selection.

☒ Life sciences ☐ Behavioural & social sciences ☐ Ecological, evolutionary & environmental sciences

For a reference copy of the document with all sections, see nature.com/documents/nr-reporting-summary-flat.pdf

Life sciences study design

All studies must disclose on these points even when the disclosure is negative.

Sample size	No sample size calculation was done in this manuscript for biology/clinical reasons, because this manuscript focuses on demonstration of methodological development. The size of the data used for showcasing methodology benchmark was determined by the size of the original dataset, unless it exceeds certain computational resource limit. In that case, reduced sample size was created by random sub-sampling.
Data exclusions	No data exclusion was done in this manuscript for biology/clinical reasons.
Replication	No replication was done in this manuscript for biology/clinical reasons. For benchmarking the algorithm, each test was performed 5 times with random subsamples of the related dataset, to maintain the robustness of the conclusion.
Randomization	No randomization was done in this manuscript for biology/clinical reasons. Control of covariates for clinical finding in this study was not

Randomization	relevant since such tasks were not pursued in this study. For benchmarking the algorithm, random sub-sampling of single cells were performed to demonstrate the performance of the algorithm on a representative cell population composition.
Blinding	No blinding was done in this manuscript for biology/clinical reasons. Blinding is not relevant for this study because the experiments were aiming to validate technical performance.

Reporting for specific materials, systems and methods

We require information from authors about some types of materials, experimental systems and methods used in many studies. Here, indicate whether each material, system or method listed is relevant to your study. If you are not sure if a list item applies to your research, read the appropriate section before selecting a response.

Materials & experimental systems

n/a	Involved in the study	
☒	☐	Antibodies
☒	☐	Eukaryotic cell lines
☒	☐	Palaeontology and archaeology
☒	☐	Animals and other organisms
☒	☐	Clinical data
☒	☐	Dual use research of concern

Methods

n/a	Involved in the study	
☒	☐	ChIP-seq
☒	☐	Flow cytometry
☒	☐	MRI-based neuroimaging
